# Single-cell spatial transcriptomic analysis reveals common and divergent features of developing postnatal granule cerebellar cells and medulloblastoma

**DOI:** 10.1186/s12915-021-01071-8

**Published:** 2021-07-01

**Authors:** Wenqin Luo, Guan Ning Lin, Weichen Song, Yu Zhang, Huadong Lai, Man Zhang, Juju Miao, Xiaomu Cheng, Yongjie Wang, Wang Li, Wenxiang Wei, Wei-Qiang Gao, Ru Yang, Jia Wang

**Affiliations:** 1 State Key Laboratory of Oncogenes and Related Genes, Renji-Med X Clinical Stem Cell Research Center, Ren Ji Hospital, School of Medicine, Shanghai Jiao Tong University, 160 Pujian Rd., Shanghai, 200127 China; 2 School of Biomedical Engineering, Shanghai Jiao Tong University, Shanghai, 200030 China; 3 Shanghai Mental Health Center, Shanghai Jiao Tong University School of Medicine, Shanghai, 200030 China; 4 School of Biomedical Engineering & Med-X Research Institute, Shanghai Jiao Tong University, Shanghai, 200030 China; 5 Department of Cell Biology, School of Medicine, Soochow University, Suzhou, 215123 China

**Keywords:** Cerebellum, Development of granule cells, Granule neuron progenitors, Differentiated granule neurons, SHH medulloblastoma, Single-cell RNA sequencing, Spatial transcriptomics

## Abstract

**Background:**

Cerebellar neurogenesis involves the generation of large numbers of cerebellar granule neurons (GNs) throughout development of the cerebellum, a process that involves tight regulation of proliferation and differentiation of granule neuron progenitors (GNPs). A number of transcriptional regulators, including *Math1*, and the signaling molecules Wnt and Shh have been shown to have important roles in GNP proliferation and differentiation, and deregulation of granule cell development has been reported to be associated with the pathogenesis of medulloblastoma. While the progenitor/differentiation states of cerebellar granule cells have been broadly investigated, a more detailed association between developmental differentiation programs and spatial gene expression patterns, and how these lead to differential generation of distinct types of medulloblastoma remains poorly understood. Here, we provide a comparative single-cell spatial transcriptomics analysis to better understand the similarities and differences between developing granule and medulloblastoma cells.

**Results:**

To acquire an enhanced understanding of the precise cellular states of developing cerebellar granule cells, we performed single-cell RNA sequencing of 24,919 murine cerebellar cells from granule neuron-specific reporter mice (*Math1-GFP*; *Dcx-DsRed* mice). Our single-cell analysis revealed that there are four major states of developing cerebellar granule cells, including two subsets of granule progenitors and two subsets of differentiating/differentiated granule neurons. Further spatial transcriptomics technology enabled visualization of their spatial locations in cerebellum. In addition, we performed single-cell RNA sequencing of 18,372 cells from *Patched*^+/−^ mutant mice and found that the transformed granule cells in medulloblastoma closely resembled developing granule neurons of varying differentiation states. However, transformed granule neuron progenitors in medulloblastoma exhibit noticeably less tendency to differentiate compared with cells in normal development.

**Conclusion:**

In sum, our study revealed the cellular and spatial organization of the detailed states of cerebellar granule cells and provided direct evidence for the similarities and discrepancies between normal cerebellar development and tumorigenesis.

**Supplementary Information:**

The online version contains supplementary material available at 10.1186/s12915-021-01071-8.

## Background

Cerebellar granule neuron progenitors (GNPs) are an excellent model to study neuronal proliferation and differentiation regulation in brain development. GNPs proliferate extensively during the first two postnatal weeks in the external granule layer (EGL), a transient secondary germinal zone [1–4]. The cells then exit the cell cycle and migrate inward to the internal granule layer (IGL), where they differentiate into mature granule neurons (GNs). Numerous studies have reported many regulatory factors, including transcription factors (TFs) and environmental cues, that are involved in GN proliferation, migration, and differentiation [2, 5, 6]. For example, *Math1* is the most well-known TF that is expressed transiently in the EGL at early developing stages and is required for the transit amplification of GNPs [7, 8].

Control of neuronal progenitor cell proliferation is essential for healthy brain development, and deregulation of this fundamental developmental event contributes to brain cancer [9]. Medulloblastoma (MB) is one of the most common pediatric brain tumors, and it arises from deregulated cerebellar development [10, 11]. The current knowledge based on gene expression analysis has identified four major molecular subgroups of MB, namely WNT-MB, Sonic hedgehog (SHH)-MB, group 3 MB, and group 4 MB [12, 13]. The cellular origins of MB have been investigated for many years through cell lineage-tracing strategies [14, 15]. Recently, several groups used single-cell genomics approaches to correlate the diverse MB phenotypes with the putative cells of origin. These reports demonstrated that each subtype of MB is derived from a specific cell of origin [16, 17], which thereby determines the clinical and molecular behavior of the disease. For instance, the SHH-MB group, comprising about 30% of all MBs, is demonstrated to originate from granule progenitors [18].

Single-cell RNA sequencing (scRNA-seq) has facilitated the transcriptional cataloging of cell types in both developing cerebellum [19, 20] and MB [16, 17]. However, these single-cell studies did not examine the detailed cellular states associated with the progenitor/differentiation programs of GNs. In addition, single-cell sequencing involves a preprocessing step of tissue dissociation, which leads to the loss of spatial information. Recent advances in spatial transcriptomics (ST) have overcome this challenge, although this approach is still limited in providing single-cell resolution [21].

**Fig. 1:**
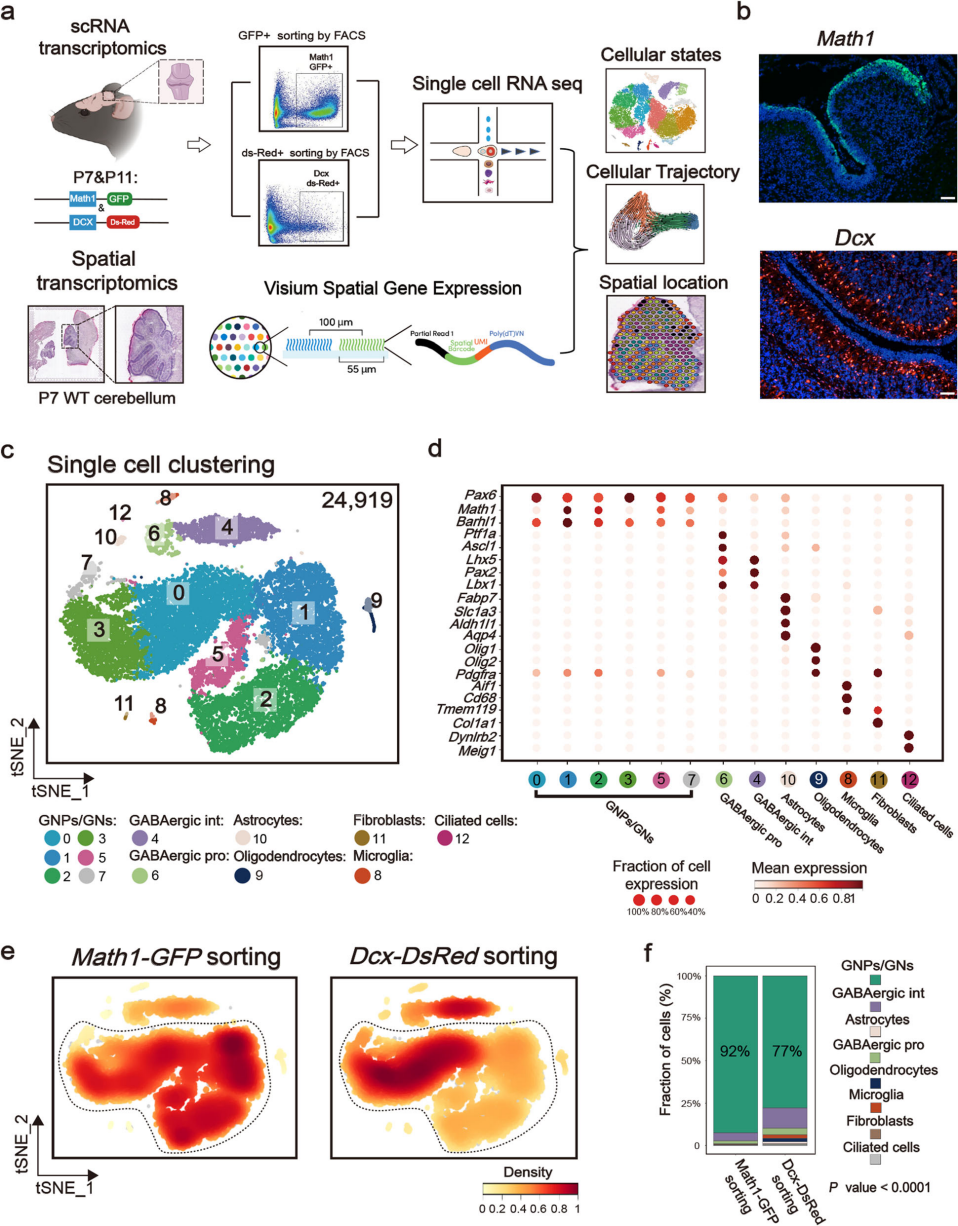
Single-cell transcriptome profiling of postnatal cerebellar cells. **a** Workflow (top panel) for cerebellum collection by FACS-sorted, single-cell sequencing, and analysis of two samples [one at postnatal days 7 (P7) and the other at P11] from *Math1-GFP* mice and two samples [one at P7 and the other at P11] from *Dcx-DsRed* mice. Workflow (bottom panel) for cerebellum section, spatial transcriptomes, and analysis of two samples from WT mice (both at P7). **b** Strains were established from transgenic *Math1-GFP* and transgenic *Dcx-DsRed* mice. Scale bar: 100 μm. **c** t-SNE visualization of 24,919 cells from FACS-sorted samples (*n* = 4; *Math1-GFP* mice at P7 and P11; *Dcx-DsRed* mice at P7 and P11). Cells are colored according to clusters with annotation of cell types. **d** Dot plot for the expression of marker genes in each cell type. Color represents the mean expression in each cell cluster, and size indicates the fraction of cells expressing marker genes. **e** t-SNE visualization of 24,919 cells from FACS-sorted samples: *Math1-GFP*^+^ and *Dcx-DsRed*^+^ samples. **f** Bar plot showing the proportion of cell types in *Math1-GFP*^+^ and *Dcx-DsRed*^+^ samples. *P* < 0.0001. *P* values were determined using Pearson’s chi-square test

Here, our study focused on developing GNs, with the aim to elucidate their cellular states and lineage trajectories. We applied both scRNA-seq and ST techniques (Fig. 1a) to profile the cellular subpopulation and associated spatial locations in early postnatal mouse cerebellum. Additionally, we performed scRNA-seq for three MB tumors developed in *Patched*^+/−^ mutant mice and acquired the single-cell transcriptome of tumor cells. We then performed the comparative analyses to assess the similarity and differences between normal developing GNs and tumor cells. These findings will facilitate a broader functional understanding of the progenitor/differentiation balance as well as relevance to MB tumorigenesis.

## Results

### Single-cell transcriptome profiling of postnatal cerebellar cells

We have previously used the *Math1*-*GFP* and *Dcx*-*DsRed* reporter mouse lines to study the cell division modes of developing GNs [22]. As shown in Fig. 1b, the GFP^+^ high cells represented *Math1*-expressing GNPs, while red fluorescent-labeled cells corresponded to the *Dcx*-expressing differentiating GNs. In this study, to focus on GN development, we performed scRNA-seq for both *Math1-GFP*^+^ and *Dcx-DsRed*^+^ cells isolated from postnatal cerebellum tissues of transgenic mice. Fluorescent-labeled cells were isolated using fluorescent-activated cell sorting (FACS) from the early postnatal cerebellum at postnatal day 7 and 11 (P7 and P11), as GN cells undergo massive proliferation and differentiation during the first 2 weeks after birth [1, 3].

After quality control including doublet removal, we obtained a total of 24,919 FACS-purified cells. Unsupervised clustering using t-distributed stochastic neighbor embedding (t-SNE) revealed thirteen clusters (Fig. 1c). Based on the known annotations of marker genes from the literature [16, 19, 23], we divided these cell populations into eight cell lineages, including GNP/GN cells (expressing *Math1*, *Barhl1* and *Pax6*), GABAergic progenitors (GABAergic pro) (expressing *Ptf1a*, *Ascl1*, and *Lhx5*), GABAergic interneurons (GABAergic int) (expressing *Pax2* and *Lbx1*), astrocytes (expressing *Fabp7*, *Slc1a3*, *Aldh1l1*, and *Aqp4*), oligodendrocytes (expressing *Olig1*, *Olig2*, and *Pdgfra*), fibroblasts (expressing *Col1a1*), microglia (expressing *Aif1*, *Cd68*, and *Tmem119*), and ciliated cells (expressing *Dynlrb2* and *Meig1*) (Fig. 1d). We found that the proportion of GNPs/GNs in *Math1-GFP*^+^ sorted cells was significantly larger than in *Dcx-DsRed*^+^ sorted cells (92% in *Math1-GFP*^+^ cells, 77% in *Dcx-DsRed*^+^ cells, *P* < 0.0001, Pearson’s chi-square test; Fig. 1e, f), in line with the fact that *Dcx* is broadly expressed in both GNs and other migrating neurons [24].

Considering that the biased FACS-based sorting strategy may result in the loss of some cell populations/states of developing GN cells, we also collected whole cerebellar cells from wild-type (WT) mice for scRNA-seq (Additional file 1: Figure S1a). We obtained 20,367 cells from two WT samples, which were then integrated with cells isolated from *Math1-GFP*^+^ and *Dcx-DsRed*^+^ reporter mice (45,286 cells) (Additional file 1: Figure S1b). This analysis showed two additional cell types, Purkinje cells (PCs, expressing *Calb1* and *Car8*) and unipolar brush cells (UBCs, expressing *Eomes*) in WT cerebellar cells (Additional file 1: Figure S1f). Additionally, astrocytes (expressing *Fabp7*, *Slc1a3*, *Aldh1l1*, and *Aqp4*) were also mainly derived from unsorted samples (Additional file 1: Figure S1d and S1e). Notably, there was no unique granule neuron assigned to WT samples, suggesting that our FACS strategy did not result in the loss of any GN subsets (Additional file 1: Figure S1d and S1e). Nevertheless, probably due to a high proportion of granule neurons in developing cerebella, the lineage-tracing strategy in our study did not show an obvious advantage over WT samples in enriching granule neurons for single-cell sequencing.

### Identifying distinct states associated with postnatal GN development

**Fig. 2:**
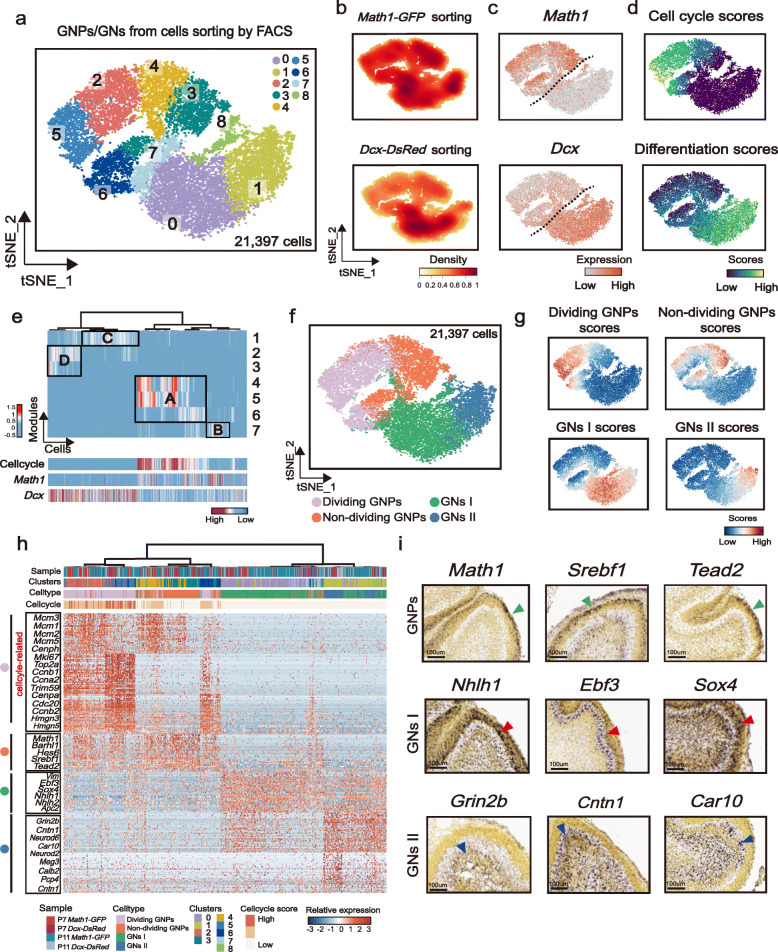
Identifying distinct states associated with postnatal GN development. **a** t-SNE visualization of 21,397 granule neuron cells (GNs) from FACS-sorted samples after re-clustering. *n* = 4 mice. They are Math1-GFP mice at P7 and P11, as well as Dcx-DsRed mice at P7 and P11. Cells are colored according to clusters. **b** t-SNE visualization of FACS-sorted sample sources including *Math1-GFP*^+^ and *Dcx-DsRed*^+^ samples. **c** Signature gene expression of GNPs (*Math1*) and differentiating/differentiated GNs (*Dcx*). **d** t-SNE visualization of cell cycle and differentiation (*Rbfox3*, *Grin2b*, and *Neurod1*) gene scores. **e** Scores of GNs (X-axis) for seven modules (Y-axis) derived from Monocle 3 module analysis. Four highly correlated modules are highlighted (modules A, B, C, and D). Cells and modules are hierarchically clustered. Scores of cell cycle genes, and expression of *Math1* and *Dcx* are ordered as on the top. **f** Four cell states are defined corresponding to the four main modules in **e**. **g** Scores of the four main module genes are shown. **h** Heatmap depicting gene expression levels of markers in the four GN states, with color-coding for the corresponding FACS-sorted sample, clusters, cell types, and cell cycle scores. **i** Signature gene expression of GNPs (Math1, Srebf1, and Tead2), GNs I (Nhlh1, Ebf3, and Sox4), and GNs II (Grin2b, Cntn1, and Car10). In situ hybridization (ISH) data were obtained from the Allen Developing Mouse Brain Atlas (© 2008 Allen Institute for Brain Science. Allen Developing Mouse Brain Atlas http://developingmouse.brain-map.org). Scale bar: 100 μm. Mouse cerebellum at P4 are shown

The progenitor/differentiation states of GNs have been characterized by many previous studies, which suggested that there are at least two major cell subsets of GN cells referred as GNPs and GNs that differ in cell cycle activities and spatial locations [25]. To obtain a better understanding of the cellular states associated with developing postnatal GN cells, we computationally isolated and re-clustered GNPs/GNs of *Math1-GFP*^+^ and *Dcx-DsRed*^+^ cells, which revealed nine sub-clusters (Fig. 2a). Five clusters (cluster 2, 3, 4, 5, and 6) highly expressed the progenitor cell marker *Math1* (Fig. 2c), while the remaining clusters (cluster 0, 1, 7, and 8) showed upregulated expression of *Dcx* (Fig. 2c), which corresponds to differentiating/differentiated cell states. GNPs have been shown to possess high proliferation potential. In keeping with this notion, most *Math1*-expressing cells showed markedly unregulated cell cycle activities (Fig. 2d). In contrast, *Dcx*-expressing cells showed downregulated cell cycle activities and an upregulated gene set that was previously used to highlight the differentiating/differentiated GNs (*Rbfox3*, *Grin2b*, and *Neurod1*) [23, 26] (Fig. 2d). As expected, *Dcx*-expressing cells were largely derived from *Dcx-DsRed*^+^ sorted samples (Fig. 2b). In contrast, *Math1-GFP*^+^ sorted strategy seems not to be able to enrich GNPs. We speculate that this is due to our FACS threshold that was set for collecting all *Math1*-expressing cells but not for *Math1*^high^ GNPs (Additional file 1: Figure S1c).

To gain a global understanding of the undifferentiated/differentiated states of GNs, we next applied Moran’s I test implemented in Monocle 3 [27, 28] to dissect the underlying transcriptional modules. This analysis identified seven modules differentially expressed within and among Louvain component clusters in Monocle 3 (Fig. 2e, Y-axis). All GNPs/GNs were then scored with representative genes of each module (Fig. 2e, X-axis). By hierarchical clustering and subsequent gene ontology (GO) enrichment analysis, these seven modules were further grouped into four main modules (Additional file 4: Table S3) that may correspond to four distinct states of GN cells (Fig. 2e, Additional file 1: Figure S2a). Module A contained many genes correlated with nuclear division and cell cycle (Additional file 4: Table S3), corresponding to the dividing *Math1*-expressing GNPs (Fig. 2e). Module B was highly expressed in a small population of *Math1*-expressing GNPs with downregulated cell cycle activities, likely corresponding to the non-dividing GNPs (Fig. 2e). In contrast, modules C and D characterized by gene ontology as associated with neurogenesis and neuron differentiation were highly upregulated in *Dcx*-expressing non-cycling cells, indicating that there were likely two subsets of differentiated GNs (Fig. 2e). We next scored individual cells according to these four modules, which assigned GNPs/GNs into dividing GNPs (corresponding to module A), non-dividing GNPs (corresponding to module B), GNs I (corresponding to module C), and GNs II (corresponding to module D) (Fig. 2f, g).

We next assessed the transcriptional signature of these four states of GNs. As shown in Fig. 2 h, both dividing and non-dividing GNPs highly expressed progenitor-associated signature genes (such as *Math1*, *Barhl1*, *Hey1*, and *Hes6*), but differed in cell cycle-related gene expression. In addition to the well-known marker genes, some novel marker genes were associated with the GNP state. For example, *Srebf1* and *Tead2*, which were both introduced as critical regulators of cerebral neuron development [29, 30], were highly expressed in the progenitor state of GN. To validate this finding, we then checked their expression patterns with in situ hybridization (ISH) from the Allen Developing Mouse Brain Atlas [31]. As shown in Fig. 2i, Srebf1 and Tead2 are both expressed in the outer EGL, similar to Math1 expression.

We next turned our attention to the differentiated GNs and found that GNs I highly expressed *Vim*, *Ebf3*, *Apc2*, *Sox4*, *Nhlh2*, and *Nhlh1* (Fig. 2h). Among these markers, *Vim* has been widely linked with epithelial-mesenchymal transition and cell migration [32, 33] and *Nhlh1* is a TF linked to the onset of granule cell differentiation in migrating granule cell precursors [34]. In addition, *Nhlh2* was previously found in the premigratory zone of the EGL where GNPs undergo the initial stages of neuronal differentiation [35] and *Apc2*-deficient neurons could impair the migration of GNs [36]. These findings indicated that GNs I likely represented a group of differentiating/migrating GNs. In line with our expectations, ISH analysis revealed that the GNs I marker Nhlh1 was strongly expressed in the inner EGL (Fig. 2i). Other markers detected in GNs I, such as Sox4 and Ebf3, were also expressed in the inner EGL (Fig. 2i). GNs II highly expressed markers that have been reported to correspond to GN differentiation such as *Grin2b*, *Calb2*, and *Cntn1* [23] and a novel marker such as *Car10*. The expressions of Grin2b, Calb2, and Car10 were detected within the IGL region (Fig. 2i), suggesting that GNs II represented a population of more committed GNs. Together these findings show that the delicate cell states of developing GNs and associated signature genes were highlighted at single-cell resolution, revealing four distinct states of postnatal developing GNs. Whether some unreported molecules in developing GNs such as *Srebf1*, *Tead2*, *Vim*, *Sox4*, *Ebf3*, and *Car10* play a vital role in regulating the undifferentiated/differentiated states of GNs should be explored in future studies.

### TF regulatory networks underlying cell states of GNs

**Fig. 3:**
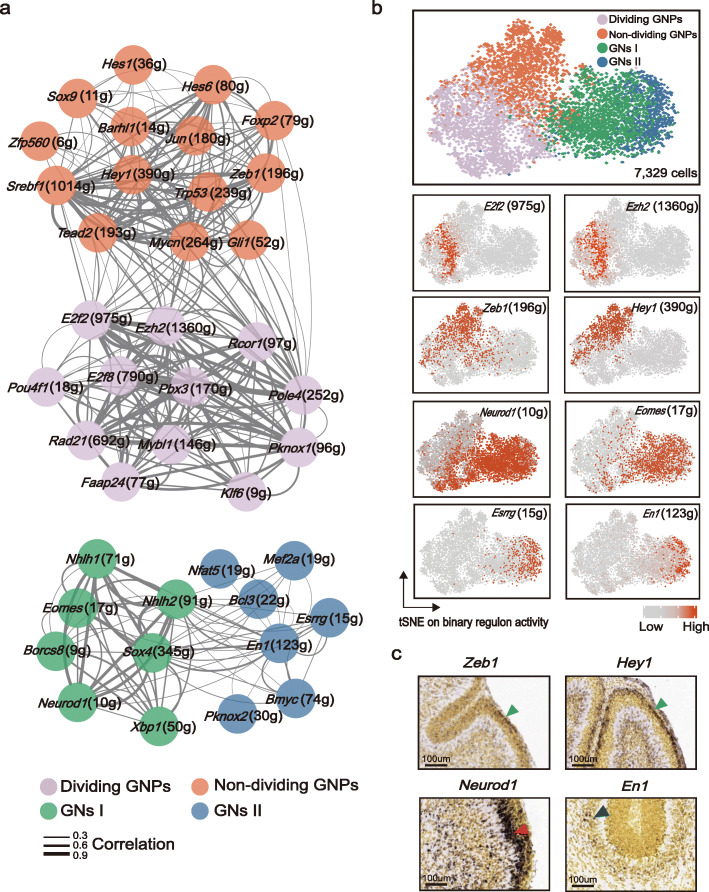
TF regulatory networks underlying cell states of GNs. **a** Network showing the correlation of regulons in four GN states. Nodes are colored according to cell types. The edge width corresponds to the values of correlation between regulons. **b** t-SNE visualization on the binary regulon activity matrix. Cells are colored according to four cell states. t-SNE visualization of regulon activities in four GN states. **c** Expression of Zeb1, Hey1, Neurod1, and En1 in the mouse cerebellum. In situ hybridization (ISH) data were obtained from the Allen Developing Mouse Brain Atlas (© 2008 Allen Institute for Brain Science. Allen Developing Mouse Brain Atlas http://developingmouse.brain-map.org). Scale bar: 100 μm. Mouse cerebellum at P4 are shown

Cell phenotypes/states are governed by core regulatory TFs that interact with cis-regulatory elements, namely regulons, for directing transcriptional programs [37]. We next utilized SCENIC [37] to identify the master TF networks associated with the four cell states in the GN cells. This result revealed that the four states of GN cells were constructed by distinct regulons (Fig. 3a). For example, as expected, the dividing GNPs are driven by many cell cycle-related regulons, including *E2f2* and *Ezh2* (Fig. 3b). Of interest, the non-dividing state of GNPs was orchestrated by a unique set of regulons, such as *Zeb1* and *Hey1* (Fig. 3b). The subsequent ISH examination confirmed that Zeb1 and Hey1 were expressed in the outer EGL (Fig. 3c). GNs I was highlighted by the regulon of *Neurod1*, which has been previously demonstrated to terminate GNP proliferation and thereby promote migration and differentiation [38]. In addition, we found that *Eomes*, the specific marker of UBCs [39], was also upregulated in GNs I (Fig. 3b). In ISH, *Neurod1*-positive cells were enriched in the inner EGL and molecular layer/Purkinje cell layer (ML/PCL), validating the migrating state of GNs I (Fig. 3c). GNs II showed significant upregulation of the *En1* regulon, which was strongly expressed in the inner EGL at E17.5 in the previous report [40]. In comparison, *En1* has dynamic expression patterns throughout cerebellar development. Examination of En1 at P4 revealed that most En1-positive cells were located in the IGL (Fig. 3c). Notably, the GNs II preferentially activated many TF regulons that have not been characterized in GN development, including *Bmyc*, *Bcl3*, and *Esrrg* (Fig. 3b). Taken together, SCENIC analysis supported that postnatal GNs displayed four distinct states.

### Identifying cell populations in the cerebellum with ST

**Fig. 4:**
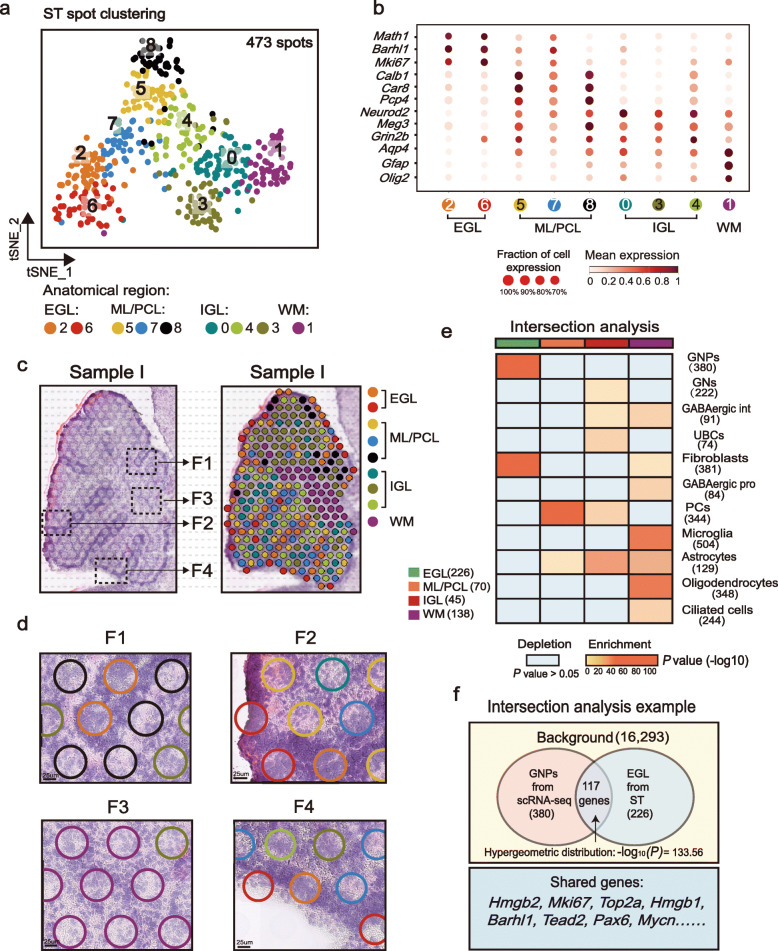
Identifying cell populations in the cerebellum with ST. **a** Dimensionality reduction and clustering of 473 spots from cerebellum section from WT mice at P7. *n* = 2 mice. They are sample I and sample II. Each cluster’s annotated anatomical region of sample I is indicated in **c**. Spots are colored according to clusters. **b** Dot plot for the expression of representative marker genes in cerebellar cell types corresponding to the anatomical region. Color represents the mean expression in each cluster, and size indicates the fraction of cells expressing the marker genes. **c–d** Mapping spots to their spatial positions shows that spots defined by marker genes are localized to the expected layers of cerebellum in sample I. Magnified images of the histological structures are shown in F1–F4. Scale bar: 25 μm. **e–f** Intersection analysis of all scRNA-seq-identified cell types and spatial transcriptomics-defined regions. Each value of the heatmap is computed as described in **f**. All pairs of cell types and cerebellum region using the same background genes (16,293 genes). The numbers of cell-type-specific and spatial region-specific genes used in the calculation are shown in **f**. Red indicates enrichment (significantly high overlap; *P* value < 0.05) and blue indicates depletion (*P* value > 0.05). The bar on the top indicates the regions defined in **a**

Although ISH data provided evidence for the spatial location of the identified GN subsets, we next used ST to capture more comprehensive spatial information of these cell types. ST was performed on brain sections from two C57BL mice at P7 (Additional file 1: Figure S3a), which generated a total of 473 individual spots on the H&E-stained slice of the developing cerebellum at P7. We then performed the dimensionality reduction and clustering analysis on the spatial (spot) gene expression profiles and identified nine sub-clusters (Fig. 4a). Based on the expression of the cell-type markers, these clusters primarily represented layer-enriched neuron populations present across the EGL, ML/PCL, and IGL to WM areas (Fig. 4b). For example, spots of cluster 2 and 6 highly expressed GNP markers such as *Mki67*, *Math1*, and *Barhl1*, corresponding to the EGL. Spots of clusters 5, 7, and 8 with strong expressions of PC marker genes (such as *Calb1*, *Pcp4*, and *Car8)* mapped to the ML/PCL. Of note, here we merged ML and PCL together because of the limited resolution of ST spot (55–100 μm). Next, we mapped the clustered spots back to their original coordinates in the cerebellar section and found that t-SNE clusters were projected into different histological annotations, supporting the ability to identify spatial regions based on ST gene expression (Fig. 4c–d, Additional file 1: Figure S3b–S3c).

To further reveal the cell types of mouse cerebellum at P7 in the spatiotemporal overview, we next applied intersection analysis [41] to compute the overlap between each pair of cell-type-specific and region-specific gene sets and performed a hypergeometric test to assess whether their overlap is significantly enrichment (*P* < 0.05) or depletion (*P* > 0.05) than expected by chance (for example see Fig. 4f; see “Methods”). The enrichment of overlap between the cell type and region is shown as a heatmap in Fig. 4e. We found that GNP and GN-specific genes in the postnatal cerebellum of our scRNA-seq data overlapped with the set of genes specific to EGL and IGL identified by ST, respectively (Fig. 4e; *P* < 0.001). For GABAergic lineage cells, GABA progenitors and interneurons were enriched in WM as previously reported [42] (Fig. 4e; *P* < 0.001), while ML/PCL were indeed enriched with PCs (Fig. 4e; *P* < 10^−10^). As expected, the WM region was also enriched with glial lineage cells [43, 44] like astrocytes and oligodendrocytes (Fig. 4e; *P* < 10^−10^). In addition, astrocytes were also enriched in ML/PCL and IGL (Fig. 4e; *P* < 0.05). Similar to what has been reported [44], it might be that there are three types of astrocytes in the murine cerebellar cortex, Bergmann glia in the PCL, fibrous astrocytes in WM, and protoplasmic astrocytes in IGL, leading to such enrichment. However, it seems that ST is unable to map the EGL location of astrocytes in developing cerebellum, as several studies have reported that there is a small population of scattered *Nestin*-expressing astrocyte progenitors within the EGL in addition to other locations (ML/PCL, IGL and WM) [45, 46]. It is possible that the high enrichment of granule cell in EGL attenuate the phenotype of astrocyte progenitor. In addition, the region of WM was also enriched with microglia, in line with the previous study [47] (Fig. 4e; *P* < 10^−10^). Fibroblasts were detected in both EGL (Fig. 4e; *P* < 10^−10^) and WM (Fig. 4e; *P* < 0.05), and ciliated cells were enriched in WM (Fig. 4e; *P* < 0.001). Taken together, nearly all cell lineages of cerebellum could be accurately positioned in ST, supplying a full characterization of the developing cerebellum at P7. By mapping cell types to ST, it makes sure that we could subset the regions corresponding to GN cells precisely for further study.

### Identification and mapping of GN cell-type subpopulations across cerebellar regions

**Fig. 5:**
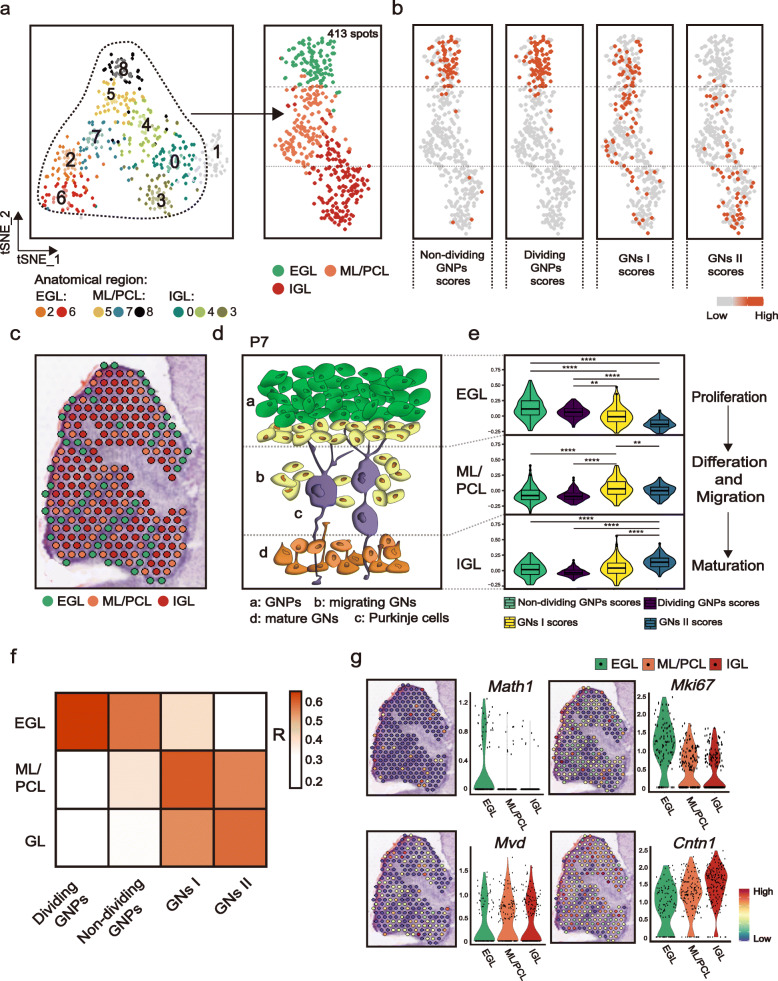
Identification and mapping of GN cell-type subpopulations across cerebellar regions. **a** t-SNE visualization of spots identified as EGL, ML/PCL, and IGL in Fig. 4a. **b** Scores of genes specifically associated with four GN states in ST spots. **c** Spatial locations of anatomical regions associated with the development of GNs: EGL, ML/PCL, and IGL. Spots are colored according to the layers. **d** Model at P7 for the cellular architecture of GNs in different development phases: a. GNPs in EGL, b. migrating GNs in the inner of EGL and ML/PCL, and d. mature GNs in IGL. c. Purkinje cells are shown in ML/PCL. **e** Violin plot for scores of selected genes corresponding to four cell states in three layers’ location. Color represents four cell states. ^*^P < 0.05; ^**^P < 0.01; ^***^P < 0.001; ^****^P < 0.0001. *P* values were determined using two-sided unpaired Wilcoxon test. **f** Correlation between four states of GNs and three layers’ location. **g** Signature gene expression of GNPs (Math1 and Mki67), GNs I (Mvd), and GNs II (Cntn1) in the ST H&E image

The EGL exclusively produces GN progenitors, which then migrate inward through the ML/PCL into IGL during early postnatal life. We further investigated subpopulations within postnatal GNs in the ST data and paid attention to the regions of EGL, ML/PCL, and IGL and discarded 60 spots defined as WM, which is mainly enriched for glial lineage cells (Fig. 5a, c). As each ST spot contains approximately 30 cells that reflect a mixed expression signature, we next filtered signature genes associated with other cell types, especially PCs within PCL and other cell types including glial cells and GABAergic lineage that may localize in ML/PCL during development (Additional file 1: Figure S4b). This step generated a total of 127 genes that are specifically associated with the GN identity (Additional file 1: Figure S4b, Additional file 6: Table S5). Using this gene set, we scored each spot (Fig. 5b, e) and found that GNPs including non-dividing GNPs and dividing GNPs were enriched in the EGL (*P* < 0.01; two-sided unpaired Wilcoxon test), while GNs I were enriched in the ML/PCL (*P* < 0.01), suggesting their migrating role during development. GNs II that are thought to be mature GNs were enriched in the IGL (*P* < 0.0001). Correlation analysis also supported the correspondence between the four states of GN cells and location of the three layers (Fig. 5f).

Next, we checked the expression levels of certain cell-type markers in the ST H&E image. As shown in Fig. 5g, markers of GNPs such as *Math1* and *Mki67* were expressed in the EGL, while *Mvd*, one of the genes specifically in GNs I, was expressed in the ML/PCL and the IGL region expressed *Cntn1*, which was similar to the outcome of ISH in Fig. 2h. Collectively, by mapping across the four cell types of scRNA-seq and the cerebellum tissue of ST data, the cell states of postnatal GN cells were described spatially and in line with previous ISH data. However, ISH shows a limitation in its dependency on available in situ probes. Therefore, the overview of cell states of GNs by ST enabled us to gain a better understanding of the expression patterns of GN cell states. Combined ISH and ST analyses confirmed that GN cells at P7 were described thoroughly as a sequential commitment from GNPs in the EGL, migrating GNs in the ML/PCL to the finally mature granule cells in the IGL, as the model shows in Fig. 5d.

### Developmental trajectories within GN lineage cells

**Fig. 6:**
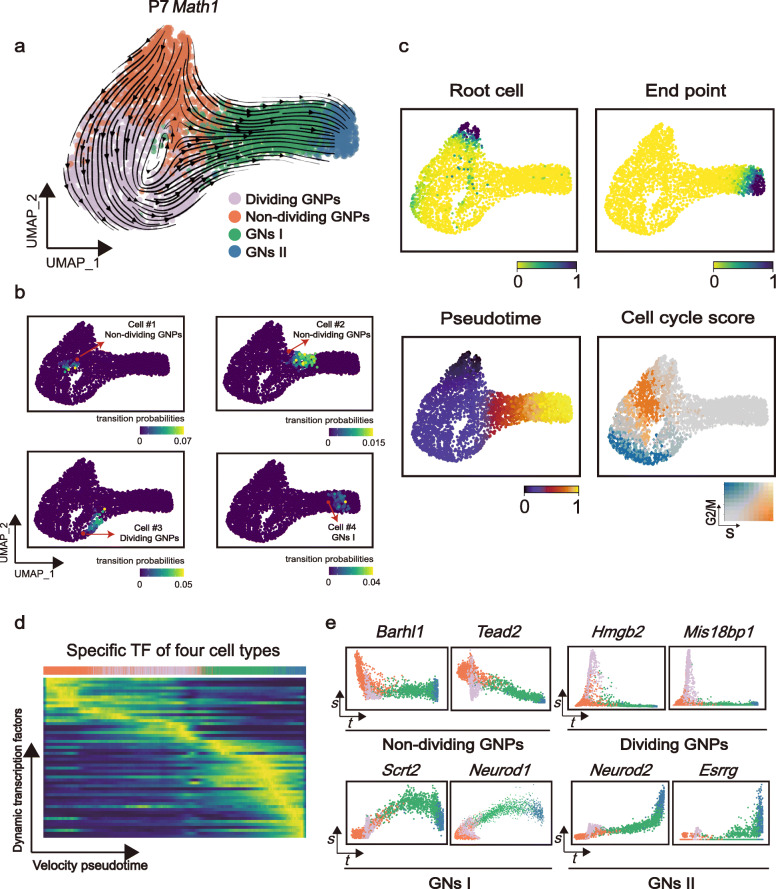
Developmental trajectories within GN lineage cells. **a** RNA velocities of GNs for *Math1-GFP*^*+*^ mouse at P7. **b** UMAP visualization of the transition probability of particular cells corresponding to four GN states. **c** The velocity-inferred root/end cells, velocity pseudotime, and cell cycle scores of P7 *Math1-GFP*^*+*^ mouse are shown. **d** Gene expression dynamics resolved along velocity pseudotime show a clear cascade of transcription of top likelihood-ranked TFs (likelihood > 0). **e** Driver genes are identified by high likelihoods. Expression dynamics along velocity pseudotime for the driver genes characterize their activity

We next sought to illuminate the lineage relationship of postnatal granule cells by performing cell trajectory analysis using scVelo [48], a new version of RNA velocity analysis [49], which applies a likelihood-based dynamical model to solve the full gene-wise transcriptional dynamics. We performed this analysis for each individual sample and observed a consistent cell differentiation trend from GNPs to differentiated GNs (Fig. 6a, Additional file 1: S5a–S5d). For example, in the P7 *Math1*-positive sample, RNA velocity predicted non-dividing GNPs as the root cells that direct cellular differentiation by two different paths (Fig. 6a, c). A substantial number of non-dividing GNPs showed differentiation tendency towards dividing GNP phase (represented by cell #1, Fig. 6a, b) and subsequently migrating GNs (represented by cell #3, Fig. 6a, b) as well as the differentiated GNs (represented by cell #4, Fig. 6a, b) in P7 *Math1*-positive sample. In contrast, there were a few non-dividing GNPs that directly differentiate into migrating GNs without undergoing the transit-amplifying state in P7 *Math1*-positive sample (represented by cell #2, Fig. 6a, b).

Besides modeling cellular dynamics, scVelo analysis also enabled us to investigate the transcriptional dynamics of variable genes during cellular state progression. We focused on the TF genes that may act as driver genes underlying cell state transition. Visualizing the ratio of unspliced to spliced mRNA abundance showed a tightly controlled expression of cell state-associated TFs (Fig. 6d). For example, the expressions of non-dividing GNP markers *Barhl1* and *Tead2* (Fig. 6e) were downregulated when cells enter the cycling progenitor state. In contrast, *Neurod1* and *Scrt2* that play a modulatory role in cortical neurogenesis and neuronal migration [50] switched at GNs I (Fig. 6e). For GNs II, *Neurod2*, which is associated with neural differentiation, started to increase in expression, and *Esrrg* switched in line with the SCENIC results (Fig. 3b). Overall, the pseudotime ordering identified by RNA velocity revealed a sequential commitment from non-dividing GNPs to dividing GNPs, migrating granule cells and the finally mature granule cells.

### Relationship between tumor cell identity and developmental GN cell origins

**Fig. 7:**
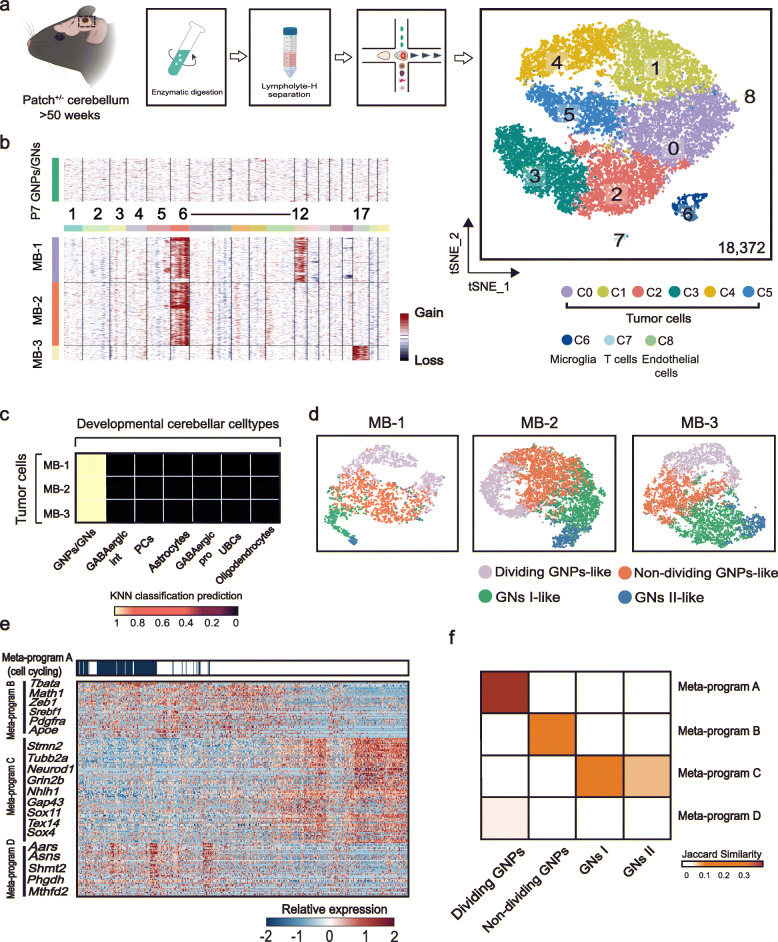
Relationship between tumor cell identity and developmental GN cell origins. **a** Workflow for the collection of MB developed after 50 weeks in *Patched*^+/−^ mice, single-cell sequencing, and clustering analysis. *n* = 3 *Patched*^+/−^ mice. They are MB-1, MB-2, and MB-3. t-SNE visualization of nine clusters in 18,372 cells from three MB tumors. Cells are colored according to clusters with annotation of cell types. **b** Heatmap showing inferred large-scale CNVs of normal cells (GNPs/GNs at P7 WT mouse) and tumor cells of three samples. **c** Heatmap of mean similarity scores between MB and developmental neural lineage cells of cerebellum. **d** t-SNE visualization of four GN cell state scores in three MB tumors. **e** Relative expression of 150 genes representing SHH-MB meta-programs from combined tumor cells. Cells positive for the cell cycle program are indicated. **f** Jaccard similarities of the gene sets between meta-programs of tumor cells (y axis) and four cell states of granule neuron cells (x axis)

Previous studies have indicated that SHH-MB may mainly originate from early postnatal GN cells [16, 51]. We next asked whether MB tumor cells exhibited similar states resembling developing GNs. To this end, we performed scRNA-seq on MB developed after 50 weeks in *Patched*^+/−^ mice. After quality control including doublet removal, we obtained a total of 18,372 cells from MB that developed in three mice (MB-1, MB-2, and MB-3) (Fig. 7a). Based on the known annotation of marker genes from the literature, we grouped cells of nine clusters into four cell types: GNP-like tumor cells (expressing *Math1* and *Grin2b*), microglia (expressing *Aif1* and *Cd68*), T cells (expressing *Cd3d* and *Cd3e*), and endothelial cells (expressing *Cldn5* and *Cdh5*) (Additional file 1: Figure S6a). We next sought to distinguish malignant cells from non-malignant cells by inferring copy-number variations (CNVs) from single-cell transcriptome profiles as described by many previous studies [52–54]. As expected, this analysis showed that GNP-like tumor cells in all three MB samples had remarkable CNVs compared with normal granule neurons at P7 (Fig. 7b), confirming their malignant identity.

Previous single-cell analyses have shown that SHH-MB phenotypically resembles developing GNs [16]. In line with this finding, *k-Nearest Neighbor* (kNN) analysis [55] predicted that the three tumors showed a transcriptional similarity to the lineage of GN cells rather than other neural cell lineages (Fig. 7c), supporting the GN origin of the three tumors. Furthermore, we compared the transcriptional similarity between tumor cells and developing cell types using Scanorama [56], which integrated tumor cell and developmental neural lineage cell-type datasets after batch effect correlation. This analysis also showed that tumor cells were transcriptionally closer to the lineage of GN cells (Additional file 1: Figure S7). Based on this observation, we next examined whether tumor cell states recapitulate the proliferation/differentiation states of normal developing GNs. For this purpose, we scored each tumor cell using the signatures of the four states of developing cells (Fig. 7d). The results revealed that the intra-tumoral states of each tumor highly resembled GN states in normal development. Further examination of signature genes associated with these four cell states supported this observation (Additional file 1: Figure S6b).

To get an unbiased understanding of intra-tumor cell states, we next applied non-negative matrix factorization (NMF) [57] to extract the underlying transcriptional programs specific to each tumor. This analysis revealed four meta-programs (A, B, C, and D) that were shared across tumors (Fig. 7e). Meta-program A was characterized by markers of the cell cycle, such as *Top2a* and *Mki67* (Additional file 7: Table S6), indicating the presence of proliferating subpopulations of tumor cells in all tumors. Meta-program B contained GNP-associated genes as mentioned above, such as *Math1* and *Zeb1*, likely corresponding to undifferentiated progenitors in each tumor. Meta-program C was defined by many markers of differentiated GNs including *Neurod1* and *Nhlh1*, probably reflecting the differentiation states of tumor cells. In addition, we identified a cancer metabolism-related meta-program (meta-program D), highlighted by *Shmt2* [58] and *Phgdh* [59] (Additional file 1: Figure S6c). To relate these meta-programs with cellular states of developing GNs, we compared their similarity by calculating Jaccard index using associated signature genes. As expected, the cell cycle meta-program A was highly similar to the dividing GNPs, while the progenitor-like meta-program B was similar to non-dividing GNPs. The differentiation-related meta-program C showed similarity to both migrating GNs and mature GNs (Fig. 7f). Moreover, the cancer metabolism meta-program D did not show a clear similarity with any states of developing GNs, suggesting that upregulation of the metabolism-associated signature may be tumor-specific. Nevertheless, our single-cell analysis demonstrated that MB has similar states with developing GNs, which provides a new angle to understand the cellular states of tumor cells.

### Delineating intra-tumoral cellular trajectories

**Fig. 8:**
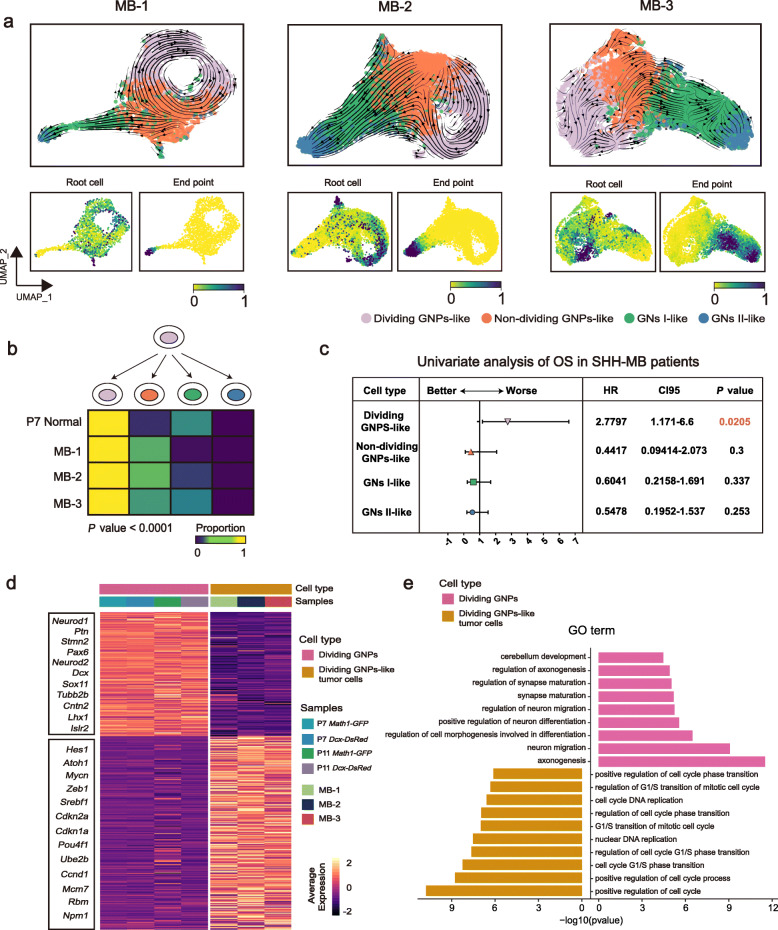
Delineating intra-tumoral cellular trajectories. **a** RNA velocities of four GN cell states like in tumor cells. **b** Heatmap showing the transition proportion from dividing GNPs and dividing GNP-like to four cell states. *P* < 0.0001. *P* values were determined using Pearson’s chi-square test. **c** Univariate analysis of overall survival (OS) in SHH-MB patients using GSVA scores of four tumor cell states. *P* values were determined using the log-rank test. **d** Heatmap of differential expressed gene analysis in dividing phase between normal and tumor models. Bar represents the average expression in each sample. **e** GO analysis of differential expressed gene in dividing phase between normal and tumor models

To further understand the intra-tumoral cellular states in our MB samples, we used scVelo to visualize the cell-cell transition trends (Fig. 8a). By computing cell-to-cell transition probabilities between different states, we found that both non-dividing GNP-like and dividing GNP-like tumor cells could serve as tumorigenic cells (root cells) in all three tumors (Fig. 8a). This is different from the normal states, in which most root cells were predicated to be non-dividing GNPs (Fig. 8a). Subsequent quantification analysis of transition probabilities supported that dividing GNP-like tumor cells exhibit higher potential to give rise to non-dividing GNP-like tumor cells rather than differentiated progeny cells especially in MB-1 and MB-2, compared with GNPs in normal developing cerebellum (*P* < 0.0001, Pearson’s chi-square test; Fig. 8b). This finding generally supported the notion that tumor progenitor-like cells displayed more self-renewal potential that impeded the differentiation process to a certain extent, which is consistent with a previous study [23].

To understand the discrepancy between normal development and tumorigenesis, we next performed gene set variation analysis (GSVA) [60] on both transformed GN and normal GN (Additional file 1: Figure S8a). As expected, we found that the SHH signaling pathway was overactivated in tumor cells especially in the progenitor-like tumor cells. In addition, other tumor-related pathways such as cell cycle, metabolism, stem cell, hypoxia, and TGF-β pathways were also upregulated in the tumor samples compared with *Math1*-positive mice at P7 and P11. Of these tumor-specific pathways, some pathways such as the tRNA aminoacylation pathway, STAT5 pathway, and ALKBH8 pathway, have been reported to contribute to cancer progression in various cancers [61–63]. We next assessed the prognostic value of these pathways and found a total of eight pathways that can predict a significantly worse outcome in the overall survival of human MB, including cell cycle, hypoxia, stem cell, tRNA aminoacylation, STAT5, and ALKBH8 pathways (Additional file 1: Figure S8b).

Finally, we considered the clinical implications of the distinct GN-like tumor populations. We therefore used signatures of four tumor cell states to score 405 human SHH-MB bulk samples. We found that patients with higher dividing GNP-like signals, whose tumors presumably contain more dividing GNP-like tumor cells, had a significant poor prognosis (*P* = 0.0205; log-rank test; Fig. 8c). This data suggested that dividing GNP-like tumor cells plays a direct role in the development of tumorigenic progression. An unbiased comparison of normal dividing GNP cells and dividing GNP-like tumor cells revealed 332 genes (Additional file 8: Table S7) that were preferentially expressed in dividing GNP-like tumor cells, including progenitor-related genes (such as *Hes1*, *Atoh1*, *Zeb1*, and *Mycn*) and cell cycle genes (such as *Cdkn2a*, *Cdkn1a*, *Pou4f1*, and *Ube2b*) (Fig. 8d). In contrast, normal dividing GNPs upregulated expression of genes related to neuron migration (such as *Neurod1* and *Dcx*) and neuron differentiation (such as *Neurod2*, *Tubb2b*, and *Pax6*), as confirmed by GO enrichment analysis (Fig. 8e). Therefore, these detailed analyses of intra-tumoral states highlighted the disparity between transformed GNs and normal developing GNs, although they shared similar undifferentiated/differentiated states. Dividing GNP-like tumor cells significantly upregulate cell cycle and progenitor-related gene programs, which to some extent inhibited the maturation of GNs.

## Discussion

Cerebellar GNs account for over half of the neurons in the brain and are mainly produced during the early postnatal stages, in which GNPs undergo extensive proliferation followed by subsequent migration and maturation. In this study, we aimed to dissect the transcriptional programs associated with postnatal granule cell development. By performing scRNA-seq for cells isolated from reporter mice (*Math1*-*GFP* and *Dcx*-*DsRed* reporter mouse lines), we constructed a single-cell transcriptomic atlas of postnatal GNs. To get a global understanding of the differentiation spectrum of granule cell development, we extracted their underlying transcriptional programs using module genes derived from Monocle 3. The results identified four discrete states of postnatal granule cells, including dividing GNPs (highly expressing cell cycle genes), non-dividing GNPs (highly expressing *Math1*, *Barhl1*, *Tead2*, and *Srebf1*), migrating GNs (expressing *Vim*, *Ebf3*, *Apc2*, *Sox4*, *Nhlh2*, and *Nhlh1*), and mature GNs (expressing *Grin2b*, *Cntn1*, *En1*, and *Car10*). We also identified several new genes that may play an important role in the development of GNs, such as *Tead2* and *Srebf1* corresponding to GNPs, *Vim*, *Sox4*, and *Ebf3* corresponding to migrating GNs and *Car10* corresponding to mature GNs. Using RNA velocity analysis, we examined the differentiation tendency between these states and found that most GNs follow a differentiation trajectory from non-dividing GNPs to dividing GNP phase and subsequently into migrating GNs and then give rise to the well-differentiated GNs. Our study thus provides more insight into the differentiation states of postnatal GNs.

However, it should be noted that the development samples in our current study did not cover the very early embryonic stages when cerebellar granule neurons are derived from the upper rhombic lip. Future studies are required to characterize the whole development process of cerebellar granule neurons and dissect their disparity from embryonic stages to postnatal stages.

While high-throughput drop-based scRNA-seq allows for the capture of distinct transcriptional states of granule cell differentiation, tissue dissociation before sequencing leads to the loss of spatial information, thus limiting our understanding of how GNPs differentiate from the EGL towards IGL. To address this limitation, we next took advantage of ST to map how GNPs differentiate from the EGL towards IGL. Although ST is still limited in providing single-cell resolution (55–100 μm resolution), we were able to visualize the spatial locations of four distinct states of GNs and confirm that GNs represent a subpopulation of differentiating/migrating GNs located between the EGL and IGL. Thus, integrating ST and scRNA-seq analyses facilitates comprehensive interrogation of GN differentiation with spatial information.

MB is the most common malignant brain tumor in children. The cell of origin of MB has recently been elucidated by single-cell transcriptional analysis, which showed that distinct MB subtypes are linked to discrete cerebellar cell subtypes in development [16, 17]. Among the distinct subtypes, SHH-MB has been shown to mimic the developmental program of GNs, which is consistent with previous lineage-tracing studies demonstrating that SHH-MB can be initiated from *Math1*-positive GN precursors [14, 15]. Our lab previously observed delayed differentiation of GNPs in *Patched*^*+/*−^ mice as evidenced by increased *Math1* and decreased *Dcx* and as evidenced by enhanced symmetrical cell division of *Math1*-positive GN precursors in *Patched*^+/−^ mice [22]. To better understand the cell fate bias between normal GNs and tumorigenic GNs, we next analyzed three MB samples from the *Patched*^+/−^ mice by scRNA-seq. We used the signatures of the four states of developing GNs to define the tumor cell states and found that MB that developed in *Patched*^+/−^ mice showed a similar composition resembling the early postnatal GN cells. Notably, RNA velocity-based analysis of the cellular differentiation trajectories revealed that transformed GNPs adopt markedly less differentiation potential. Notably, the increased progenitor-progenitor cell state transition was remarkably reproducible across the three tumor samples analyzed, thus providing insightful evidence for tumorigenesis. We further explored whether there are deterministic programs that influence cell states during tumor evolution. Our results showed that many pathways such as cell cycle, metabolism, stem cell, hypoxia, TGFB, tRNA aminoacylation, and ALKBH8 displayed significant upregulation in transformed granule cells. Further bulk sample analyses demonstrated that the oncogenic activities of newly identified pathways are strongly predictive of poor prognosis of SHH-MB patients. Together these findings suggested that advanced therapeutic strategies for targeting aggressive SHH-MBs could include inhibition of signaling pathways that maintain the undifferentiated/proliferative granule progenitor state.

## Conclusion

In conclusion, our study has depicted the detailed transcriptional profiles associated with distinct states of postnatal GNs at the single-cell level and mapped their spatial location by ST. These data thus provide an important resource for future study of cerebellar GN development. In addition, our work confirmed the main role of progenitor-like tumor cells in driving the tumorigenesis of SHH-MB, underscoring the importance of targeting this cell subpopulation as well as associated signaling circuitries in cancer therapy.

## Methods

### Animals

*Math1*-*GFP* transgenic mice were provided by Novartis. *Dcx-DsRed* mice (strain name: C57BL/6 J-Tg (*Dcx-DsRed*) 14Qlu/j, stock number; 009655) and heterozygous *Patched* (*Patched*^+/−^) mice (strain name: STOCK *Ptch1*^tm1Mps^/J, stock number: 003081) were obtained from the Jackson Laboratory. *Math1-GFP* mice, *Dcx-DsRed* mice, *Patched*^+/−^ mice, and wild-type (WT) C57BL mice were maintained in the Specific-Pathogen-Free Animal Research Centre of Renji Hospital. The animal experiments were approved by the Animal Research Ethics Committee of Renji Hospital, School of Medicine, Shanghai Jiao Tong University.

For developmental cerebellum scRNA-seq, six samples were collected, including two *Math1-GFP* ^+^ mice, respectively at P7 and P11, two *Dcx-DsRed*^+^ mice, respectively at P7 and P11 and two WT mice, respectively at P7 and P11. For developmental cerebellum ST, two WT mice at P7 were collected on one slice. For MB scRNA-seq, three samples developed in *Patched*^+/−^ mice were collected. The basic information for all samples is shown in Additional file 2: Table S1.

### Single-cell isolation and preparation of suspensions

For transgenic mice, cerebella from P7 or P11 *Math1-GFP* or *Dcx-DsRed* mice were dissected and incubated in 10–12 U/ml of activated papain solution plus 32 mg/ml of DNase in DMEMF-12 (Gibco) with rocking for 30 min at room temperature. The samples were then triturated with a fire-polished glass Pasteur pipette to generate a single-cell suspension. Two independent cell populations with GFP and DSRED fluorescence were analyzed on a BD FACSAria II cytometer (Becton Dickinson) using standard flow cytometry.

For WT and *Patched*^+/−^ mice, cerebella from P7 or P11 WT mice and MB developed after 50 weeks by *Patched*^+/−^ mice were dissected and enzymatically digested with collagenase IV (Gibco) and DNase I (Sigma) for 30 min at 37 °C with agitation. After digestion, samples were sieved through a 70-μm cell strainer and washed with 1% BSA and 2 mM EDTA in PBS. After centrifugation, single-cell suspensions were run through Lympholyte-H separation (CL5020; Cedarlane) to remove red blood cells and debris according to the manufacturer’s specifications. Pelleted cells were then re-suspended in PBS with 1% BSA and assessed for viability and size using a Countess instrument (Thermos).

### Preparation of cerebellum for ST

Two cerebellar tissues of P7 WT mice were embedded in Tissue-Tek (OCT) and snap-frozen using an isopentane/dry ice slurry. Cerebellar tissues were cryosectioned at 10-μm thickness while keeping the samples frozen. Samples were placed within the frame of capture areas on the Visium Spatial Gene Expression.

### Tissue staining and imaging

Tissue sections were fixed on the capture areas of the Visium Spatial Gene Expression using methanol. The nuclei were stained using hematoxylin, followed by eosin staining for the extracellular matrix and cytoplasm. The images of stained tissue sections were used to map the gene expression patterns back to the tissue sections.

### Visium Spatial Gene Expression library construction

The section was first permeabilized using a permeabilization enzyme. The poly-adenylated mRNA released from the overlying cells is captured by the primers on the spots. Incubation with the reverse transcription reagents produces spatially barcoded, full-length cDNA from poly-adenylated mRNA on the slide. Second Strand Mix is added to the tissue sections on the slide to initiate second strand synthesis. This is followed by denaturation and transfer of the cDNA from each capture area to a corresponding tube for amplification and library construction. After transfer of cDNA from the slide, spatially barcoded, full-length cDNA is amplified via PCR to generate sufficient mass for library construction. Enzymatic fragmentation and size selection are used to optimize the cDNA amplicon size. The final libraries contain primers used in Illumina amplification.

### Droplet-based scRNA-seq

As per the manufacturer’s protocol [64], single cells were processed through the GemCode Single Cell Platform using the GemCode Gel Bead, Chip and Library Kits (10X Genomics). Cell suspensions of each sample were run in the Chromium Controller with appropriate reagents to generate single-cell gel bead-in-emulsions for sample and cell barcoding, with a target output of ~ 5000 cells for each sample. Amplified cDNA and final libraries were evaluated on an Agilent BioAnalyzer using a High Sensitivity DNA Kit (Agilent Technologies). Libraries were pooled and sequenced on a NovaSeq 6000 (Illumina) at a depth of approximately 400 M reads per sample.

### scRNA-seq data preprocessing and quality control

Raw sequencing data were converted to FASTQ files with Illumina bcl2fastq, version 2.19.1 and aligned to the *Mus musculus* genome reference sequence (mm10). The CellRanger (10X Genomics, 2.1.1 version) analysis pipeline was used for sample demultiplexing, barcode processing, and single-cell 3′ gene counting to generate a digital gene-cell matrix from these data. The gene expression matrix was then processed and analyzed by Seurat package [65]. We performed Seurat-based filtering of cells based on the number of detected genes per cell (> 500) and the percentage of mitochondrial genes expressed (< 10%). The mitochondrial genes and ribosomal genes were also removed from the gene expression matrix. The basic information for single-cell datasets of all samples is shown in Additional file 2: Table S1.

### Filtering cell doublets

Doublets of scRNA-seq were excluded by first using *Scrublet* [66] and then estimating if our dataset included any clusters enriched for cell doublets based on the expression patterns of cell-type-specific markers.

### ST data preprocessing and quality control

Spots with fewer than 500 genes and genes expressed in fewer than 15 spots were excluded. Spots with over 10% mitochondrial gene expression were also discarded.

### Dimensionality reduction, clustering, and visualization

We used Seurat’s *NormalizeData* function with the method “LogNormalize” to normalize the feature expression measurements for each cell by the filtered expression matrix, multiplied this by a scale factor (10,000 by default) and log-transformed the result. Highly variable genes were then identified and used for the subsequent principal component analysis. Clustering was then performed using graph-based clustering and visualized using t-SNE with Seurat functions *RunTSNE*.

For the ST dataset, we first ran dimensionality reduction and clustered on the RNA expression data of ST, using the same workflow as used for scRNA-seq analysis. We then visualized the results of the clustering either in t-SNE space (with *DimPlot*) or overlaid on the image with Seurat’s *SpatialDimPlot* function.

### Cell types and anatomical region identification

For the scRNA-seq dataset, we defined sets of well-established marker genes for cell types and annotated each cell type based on their gene scores. For the ST dataset, considering cell-type-specific characteristics of anatomic layers in the developing cerebellum, we annotated each spot based on the expression of cell-type-specific genes. The detailed gene list is shown in Additional file 3: Table S2.

### Batch correcting and multiple dataset integration

For merging multiple scRNA-seq datasets of developmental samples, we applied Harmony integration [67], which has been shown to reduce technical batch effects while preserving biological variation for multiple batch integration. *RunHarmony* returns a Seurat object, updated with the corrected Harmony coordinates. The manifold was subjected to re-clustering use the corrected Harmony embeddings rather than PCs, set reduction = “harmony,” with parameters of Seurat analysis.

For comparing tumor and developing cell types using Scanorama, to eliminate batch effects among datasets, we performed batch effect correction using the “scanorama.integrate” function in the Python package Scanorama [56] with default parameter.

For ST samples, there was no need to reduce technical batch effects because of two cerebellar tissues on one slide.

### Defining cell scores

We used the AddModuleScore function in the Seurat R package to evaluate the degree to which individual cells express a certain pre-defined expression program as described previously [17, 57]. For example, we defined the states of developing GN cells by scoring for the module genes derived from Monocle 3. Single cells were assigned to different cell types/states based on the maximum expression score.

Cell cycle scores were calculated using a set of characteristic genes involved in cell cycle including 43 G1/S and 54 G2/M cell cycle genes [54].

### SCENIC analysis

We employed the python package “pySCENIC” to run SCENIC and used GRNBoost (SCENIC version 0.1.5) to run the co-expression modules. The motifs database for *Mus musculus* was downloaded from the website https://pyscenic.readthedocs.io/en/latest/. The input gene matrix is the normalized gene matrix of *Math1*-positive and *Dcx*-positive granule neurons of reporter mice at P7.

To obtain a higher confidence set of regulons and predicted regulon connections, we first identified cell-type regulons using DEGs and selected the top differential regulons of each cell type. We then computed Pearson’s correlation coefficient between regulons based on their AUCell values. Finally, the correlation of regulons was selected using a threshold of 0.3 and visualized using Cytoscape [68].

### Finding modules of co-regulated genes

We applied Monocle 3’s function “*pr_graph_test_res*” that has Moran’s I test and set *neighbor_graph* = “knn” to calculate modules differentially expressed within and among Louvain component clusters of UMAP space. We then identified genes for each module in Monocle 3 using Moran’s I threshold of 0.1 and *q*_val_ threshold of 0.05. We finally used top markers function among clusters in Monocle 3 to rank the genes by specificity and obtained the top 50 mean expression genes as representative genes of each module shown in Additional file 4: Table S3.

### RNA velocity

RNA velocities were predicted using velocyto in R program [49, 69]. Briefly, spliced/unspliced reads were annotated by velocyto.py with CellRanger (version 2.2.0), generating BAM files and an accompanying GTF; they were then saved in .loom files. The .loom files were loaded to the scvelo python pipeline using *scv.read* function and they generated count matrices for spliced and unspliced reads. Next, the count matrices were size-normalized to the median of total molecules across cells. The top 3000 highly variable genes were selected out of those that pass a minimum threshold of 10 expressed counts commonly for spliced and unspliced mRNA. Considering that the assumptions of a common splicing rate and the observation of the full splicing dynamics with steady-state mRNA levels were often violated, we used the function *recover_dynamics*, a likelihood-based dynamical model, to break these restrictions. Finally, the directional flow was visualized as single-cell velocities or streamlines in the UMAP embedding with the cell-type annotations.

To quantify the cell-state transition probability, we calculated the velocity-based cell transition matrix by the *transition_matrix()* function from scvelo, of which the element was the Pearson correlation coefficient between the velocity vector and cell-state difference vectors of the column cell. We then defined the destination of a cell by identifying the highest correlation value. We calculated the proportion of the cell-state transition of total dividing GNPs or GNP-like tumor cells towards different states of cells and presented this results using heatmap in Fig. 8b. Then Pearson’s chi-square test was performed on 4 × 4 cluster-by-cluster contingency tables to test the fate destinations of interested cell clusters.

### Determination of cell-type enrichment/depletion by interaction analysis

We first identified cell-type markers and ST region-specific markers using DEGs. DEGs in a given cell type compared with all other cell types were determined with the *FindAllMarkers* function from the Seurat package (one-tailed Wilcoxon rank sum test, *P* values adjusted for multiple testing using the Bonferroni correction). For computing DEGs, all genes were probed, provided they were expressed in at least 25% of cells or regions in either of the two populations compared and the expression difference on a natural log scale was at least 0.3. The detailed gene list of developmental cell types and ST annotated regions is shown in Additional file 5: Table S4. We then queried the significance of the overlap between ST genes and cell-type marker genes using the hypergeometric cumulative distribution, with all shared genes of ST and scRNA-seq as the background to compute *P* values. If *P* > 0.05, it was considered as cell-type depletion and shown in blue color.

### Correlation between ST and scRNA-seq

We first computed the DEGs of developing cell types shown in Figure S4a; all genes were probed provided they were expressed in at least 25% of cells, in either of the two populations compared and the expression difference on a natural log scale was at least 0.5. We then overlapped the DEGs of the four cell states in GN cells with the final selected corresponding module genes shown in Additional file 4: Table S3. Using this strategy, we can retain the genes specifically expressed in the four cell states of granule cells compared with other cell types. The detailed gene list is shown in Additional file 6: Table S5. These genes were used to compute Pearson’s correlation coefficient between the averaged cell-type profiles and ST region profiles.

### Defining ST region scores

We used the AddModuleScore function in the Seurat R package to define ST regions including EGL, ML/PCL, and IGL using genes shown in Additional file 3: Table S2 that were specifically expressed in the four cell states of GN cells.

### kNN analysis

To quantify the cell similarities between tumor cells and seven developmental neural cell types, including GNPs/GNs, GABAergic interneurons, PCs, astrocytes, GABAergic progenitors, UBCs, and oligodendrocytes, we projected the combined cell-by-gene expression matrix onto a shared low-dimensional PC space using *multiBatchPCA* function as previously described [70]. Then each expression matrix was subset to the union of all of the common genes independently detected in each dataset. We then applied the *kNN* regression model to predict a continuous outcome corresponding to developing cell types, using the *knn.reg* function (setting k = 20). The prediction scores corresponding to each cell type were then averaged to interpret the similarity between tumor cell and developing cell types.

### Inferred CNV analysis from scRNA-seq

We identified the malignant cells by inferring large-scale chromosomal CNVs in each single cell based on a moving averaged expression profiles across chromosomal intervals [52–54]. In particular, we used the GNs identified in developing cerebellum at P7 WT mice as the reference “normal” cells, and their average CNV value was subtracted from all cells. To run *inferCNV*, we applied a hidden Markov model (HMM) to predict the CNV level and implemented *inferCNV*’s i6 HMM model. The average CNV signal was estimated by averaging the CNV modification for autosomes.

### Expression programs of intra-tumoral heterogeneity

We applied NMF to extract the transcriptional programs of malignant cells of each tumor. We set the number of factors to 10 for each tumor. For each of the resulting factors, we considered the top 50 genes with the highest NMF scores as characteristics of that given factor. All single cells were then scored according to these NMF programs. Hierarchical clustering of the scores for each program using Pearson correlation coefficients as the distance metric and Ward’s linkage revealed four correlated sets of meta-programs. The gene list of the four meta-programs is shown in Additional file 7: Table S6.

### Cell cycle analysis

To identify cell cycle-positive cells, scores for the G1-S and G2-M phases of the cell cycle were computed. Data-derived thresholds of 2 MADs above the median were used to binarize cells into cycling and non-cycling in Fig. 7e.

### Jaccard similarity analysis

The Jaccard similarity coefficient was calculated for comparing the transcriptional similarity between two cell types using their signature genes. We evaluated the transcriptional similarity between the four meta-programs of malignant cells and signatures of four cell types/states of GN cells by calculated Jaccard similarity coefficients using the top 50 marker genes of meta-programs shown in Additional file 7: Table S6 and corresponding module genes in Additional file 3: Table S2 of the four cell states.

### Gene set variation analysis (GSVA)

We quantified the gene signatures of single cells by applying the single-sample GSVA (ssGSVA), which calculated the signature enrichment scores of individual single cells independently using a normalized matrix. We first merged scRNA-seq samples including three tumor samples and two developmental samples (*Math1*-positive at P7 and P11) using *RunHarmony.* We then performed analysis on a set of 5690 *Mus musculus* pathway signatures (MsigDB, C2 sets). The selected pathways all have a mean score difference | > = 0.1| between tumor samples and developmental samples.

### Survival analysis

The human MB dataset (GEO: GSE124814) [71] was used to evaluate the prognostic performance of GSVA scores of four cell states and pathway signatures. We dichotomized the low and high groups by the median of GSVA scores. For univariable analyses, we used the Cox proportional hazards model implemented in the R package of four tumor cell states and Kaplan–Meier survival curves of pathways were drawn and compared among subgroups using log-rank test.

### Statistical analysis

Statistical analysis was performed using R (version 4.0.0) and GraphPad Prism (version 7.04). The two-sided unpaired Wilcoxon test, Pearson’s chi-square test, and log-rank test were used in this study. Detailed descriptions of statistical tests are specified in the “Results” section and in the figure legends.

## Supplementary Information

**Additional file 1: Figure S1.** Single-cell transcriptome profiling of postnatal cerebellar cells, related to Fig. 1. (a) Workflow for cerebellum collection, single-cell sequencing and analysis of WT mice (one at P7 and the other at P11). (b) t-SNE visualization of 45,286 cells from four FACS-sorted samples integrated with two WT samples. Cells are colored according to clusters with annotation of cell types. (c) FACs data of *Math1-GFP*^+^, *Dcx-DsRed*^+^ strategies. (d) t-SNE visualization of 45,286 cells from FACS-sorted and WT samples (*Math1-GFP*^+^, *Dcx-DsRed*^+^ and WT cerebellum (CB)). (e) Left panel: Bar plots showing proportion of cell types in *Math1-GFP*^+^, *Dcx-DsRed*^+^ and WT samples. *P* < 0.0001. *P* values were determined using Pearson’s chi-square test. Right panel: Bar plot showing proportion of eight GNPs/GNs sub-clusters in *Math1-GFP*^+^, *Dcx-DsRed*^+^ and WT samples. (f) Signature genes scores of UBCs (*Eomes*) and PCs (*Car8* and *Calb1*) in FACS-sorted samples. **Figure S2.** Identifying distinct states associated with postnatal GN development, related to Fig. 2. (a) Bar plots showing the results of gene ontology (GO) enrichment analysis for four main modules derived from Monocle 3. **Figure S3.** Identifying cell populations in the cerebellum with ST, related to Fig. 4. (a) Two cerebellar tissues of P7 WT mice were embedded in one slice for ST. (b) Mapping spots to their spatial positions shows that spots defined by marker genes are localized to the expected layers of cerebellum of sample II. Magnified images of the histological structures are shown in F5–F7. Scale bar: 25 μm. **Figure S4.** Identification and mapping of GN cell-type subpopulations across cerebellar regions, related to Fig. 5. (a) A more comprehensive classification of cell types in all samples. Cluster 3 is GNs I. (b) Feature plots of GNs I–specific genes. **Figure S5.** Developmental trajectories within GN lineage cells, related to Fig. 6. RNA velocities and the velocity-inferred root/end cells of *Math1-GFP*^+^ sorted samples at P11 (b); *Dcx-DsRed*^+^ sorted samples at P7 (a) and P11 (c); and WT CB at P7 (d). **Figure S6.** Relationship between tumor cell identity and developmental GN cell origins, related to Fig. 7. (a) Scores of signature genes for GNP-like tumor cells (*Math1* and *Grin2b*), T cells (*Cd3d* and *Cd3e*), endothelial cells (*Cldn5* and *Cdh5*) and microglia (*Aif1* and *Cd68*) in 18,372 MB cells. (b) Scores of signature genes for non-dividing GNP-like (*Math1*, *Srebf1* and *Tead2*), dividing GNP-like (*Mki67* and *Top2a*), GNs I-like (*Vim*, *Sox4*, *Nhlh1* and *Nhlh2*) and GNs II-like (*Grin2b*, *Cntn1* and *Car10*) in three MB samples. (c) Bar plots showing results of gene ontology enrichment (GO) analysis for NMF meta-program A to D. **Figure S7.** Comparison between tumor cell and developmental cell types using Scanorama. t-SNE visualization of integration of tumor cells with developmental cell types using Scanorama. Cells are colored according to clusters with annotation of cell types. **Figure S8.** GSVA analysis of four cell states between normal and tumor models. (a) GSVA enrichment scores of pathway signatures in four cell states between normal and tumor models. (b) Kaplan-Meier survival curve of overall survival (OS). *P* values were determined using the log-rank test. Red indicates *P* value < 0.05.

**Additional file 2: Supplementary Table S1.** Basic information for single cell and spatial transcriptomics datasets of developmental and medulloblastoma samples. Related to Figs. 1, 4 and 7.

**Additional file 3: Supplementary Table S2.** Cell type markers. Related to Figs. 1, 4 and 7.

**Additional file 4: Supplementary Table S3.** Monocle 3 module genes. Related to Fig. 2.

**Additional file 5: Supplementary Table S4.** DEGs of cell types in developmental single cell dataset, DEGs of regions in spatial transcriptomics dataset. Related to Figs. 1 and 4.

**Additional file 6: Supplementary Table S5.** Genes specifically associated with four GN states. Related to Fig. 5.

**Additional file 7: Supplementary Table S6.** NMF program genes. Related to Fig. 7.

**Additional file 8: Supplementary Table S7.** DEGs between normal dividing GNPs and dividing GNP-like tumor cells. Related to Fig. 8.

## Data Availability

All data generated or analyzed during this study are included in this published article, its supplementary information files, and publicly available repositories. Single-cell RNA-seq and ST datasets are available in the Short Read Archive under accession number GSE156633 [72]. Code is uploaded to https://github.com/Luowenqin907/scRNA-seq.

## Abbreviations

GNs: Granule neurons
GNPs: Granule neuron progenitors
MB: Medulloblastoma
scRNA-seq: Single-cell RNA sequencing
ST: Spatial transcriptomics
EGL: External granule layer
IGL: Internal granule layer
TFs: Transcription factors
SHH: Sonic hedgehog
GABAergic pro: GABAergic progenitors
GABAergic int: GABAergic interneurons
PCs: Purkinje cells
UBCs: Unipolar brush cells
ISH: In situ hybridization
ML/PCL: Molecular layer/Purkinje cell layer
kNN: k-Nearest Neighbor
NMF: Non-negative matrix factorization
GSVA: Gene set variation analysis
