# Supplementary figures and images for: Single-cell spatial transcriptomic analysis reveals common and divergent features of developing postnatal granule cerebellar cells and medulloblastoma
Source: BMC Biol. 2021 Jul 1;19:135. doi: 10.1186/s12915-021-01071-8 (PMC8247169; doi:10.1186/s12915-021-01071-8)

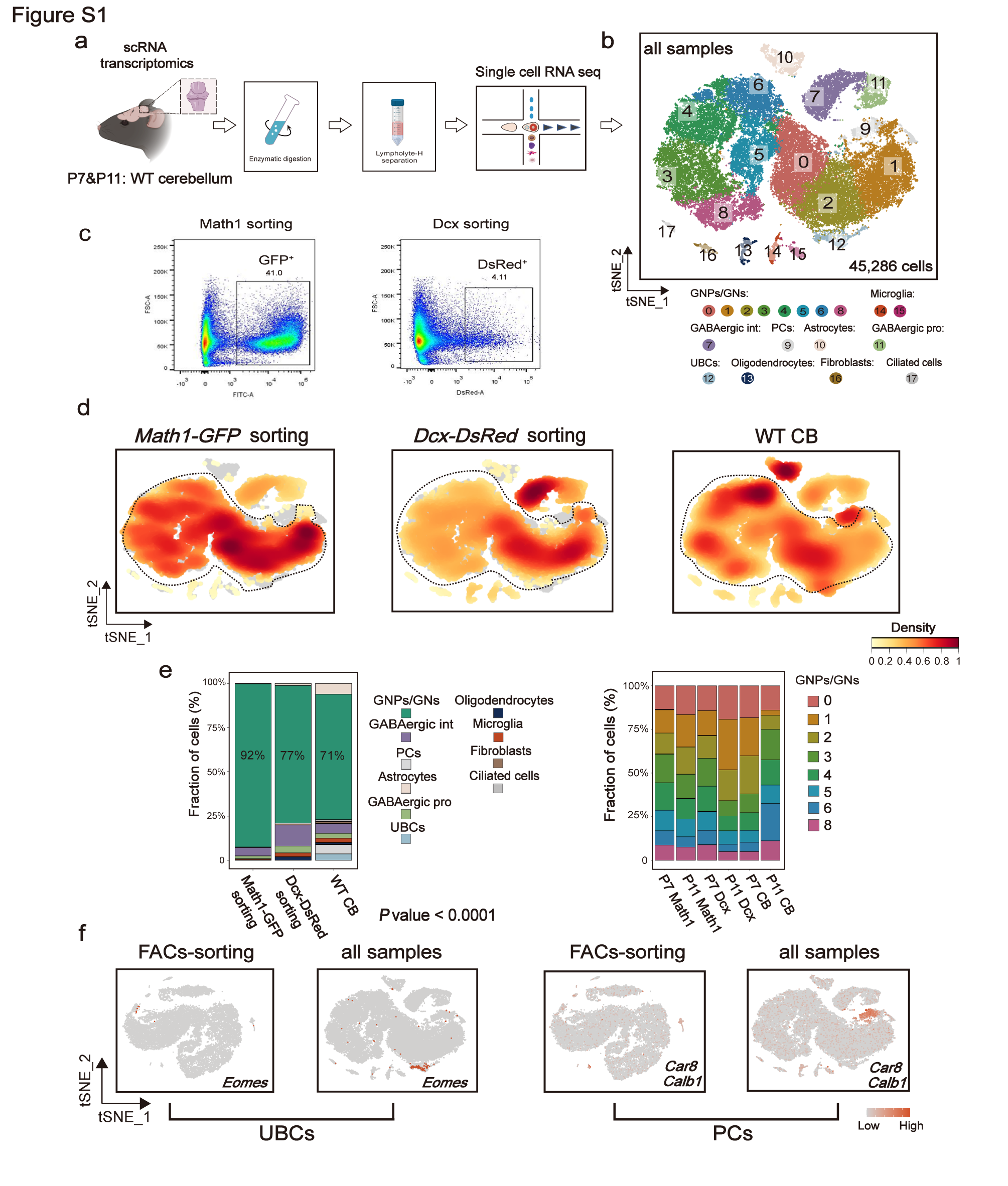

Supplement: Supplementary file 1 — Additional file 1: Figure S1. Single-cell transcriptome profiling of postnatal cerebellar cells, related to Fig. 1. (a) Workflow for cerebellum collection, single-cell sequencing and analysis of WT mice (one at P7 and the other at P11). (b) t-SNE visualization of 45,286 cells from four FACS-sorted samples integrated with two WT samples. Cells are colored according to clusters with annotation of cell types. (c) FACs data of Math1-GFP+, Dcx-DsRed+ strategies. (d) t-SNE visualization of 45,286 cells from FACS-sorted and WT samples (Math1-GFP+, Dcx-DsRed+ and WT cerebellum (CB)). (e) Left panel: Bar plots showing proportion of cell types in Math1-GFP+, Dcx-DsRed+ and WT samples. P < 0.0001. P values were determined using Pearson’s chi-square test. Right panel: Bar plot showing proportion of eight GNPs/GNs sub-clusters in Math1-GFP+, Dcx-DsRed+ and WT samples. (f) Signature genes scores of UBCs (Eomes) and PCs (Car8 and Calb1) in FACS-sorted samples. Figure S2. Identifying distinct states associated with postnatal GN development, related to Fig. 2. (a) Bar plots showing the results of gene ontology (GO) enrichment analysis for four main modules derived from Monocle 3. Figure S3. Identifying cell populations in the cerebellum with ST, related to Fig. 4. (a) Two cerebellar tissues of P7 WT mice were embedded in one slice for ST. (b) Mapping spots to their spatial positions shows that spots defined by marker genes are localized to the expected layers of cerebellum of sample II. Magnified images of the histological structures are shown in F5–F7. Scale bar: 25 μm. Figure S4. Identification and mapping of GN cell-type subpopulations across cerebellar regions, related to Fig. 5. (a) A more comprehensive classification of cell types in all samples. Cluster 3 is GNs I. (b) Feature plots of GNs I–specific genes. Figure S5. Developmental trajectories within GN lineage cells, related to Fig. 6. RNA velocities and the velocity-inferred root/end cells of Math1-GFP+ sorted samples at P [file 12915_2021_1071_MOESM1_ESM.zip › Fig S1 revised.tif]

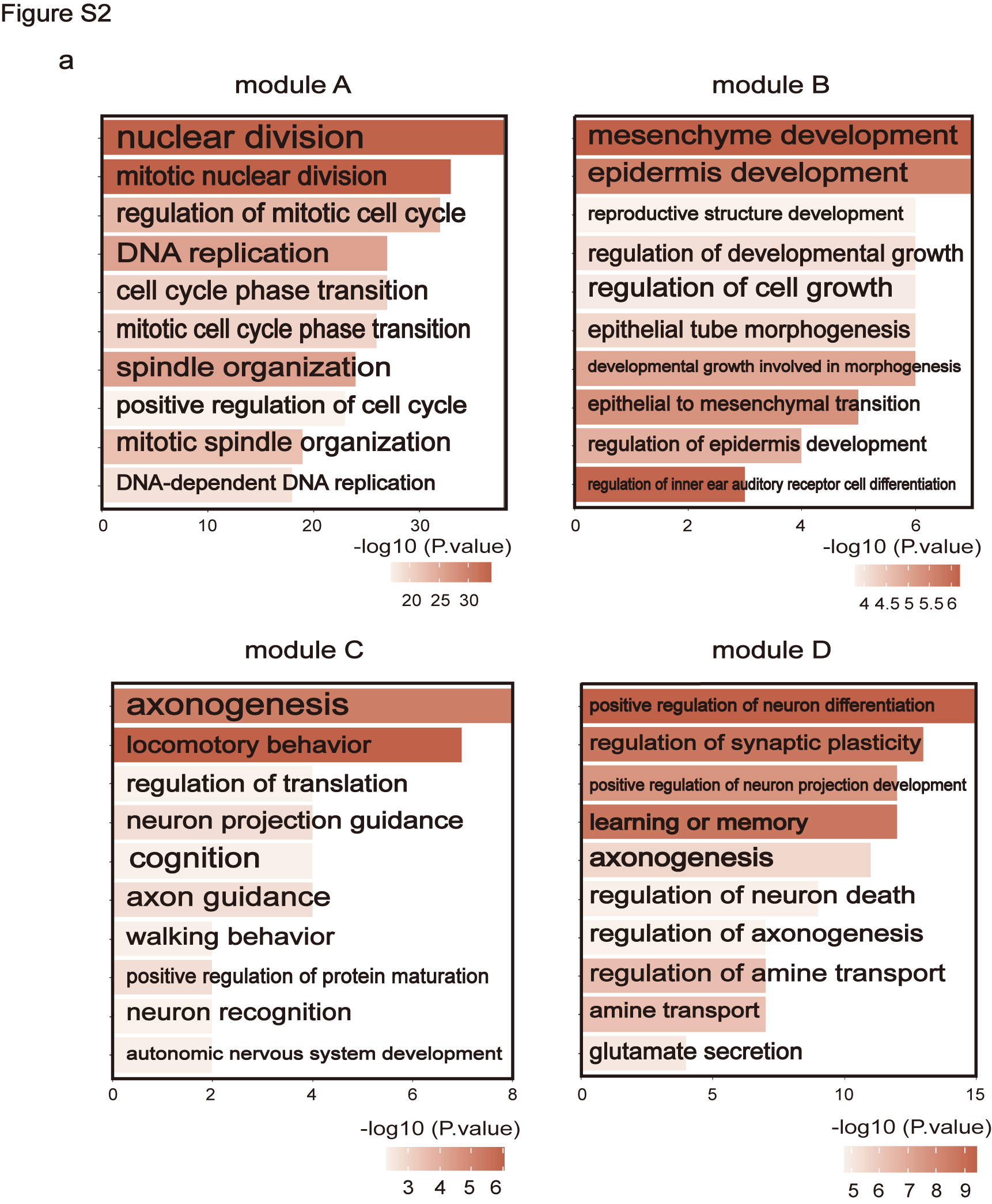

Supplement: Supplementary file 1 — Additional file 1: Figure S1. Single-cell transcriptome profiling of postnatal cerebellar cells, related to Fig. 1. (a) Workflow for cerebellum collection, single-cell sequencing and analysis of WT mice (one at P7 and the other at P11). (b) t-SNE visualization of 45,286 cells from four FACS-sorted samples integrated with two WT samples. Cells are colored according to clusters with annotation of cell types. (c) FACs data of Math1-GFP+, Dcx-DsRed+ strategies. (d) t-SNE visualization of 45,286 cells from FACS-sorted and WT samples (Math1-GFP+, Dcx-DsRed+ and WT cerebellum (CB)). (e) Left panel: Bar plots showing proportion of cell types in Math1-GFP+, Dcx-DsRed+ and WT samples. P < 0.0001. P values were determined using Pearson’s chi-square test. Right panel: Bar plot showing proportion of eight GNPs/GNs sub-clusters in Math1-GFP+, Dcx-DsRed+ and WT samples. (f) Signature genes scores of UBCs (Eomes) and PCs (Car8 and Calb1) in FACS-sorted samples. Figure S2. Identifying distinct states associated with postnatal GN development, related to Fig. 2. (a) Bar plots showing the results of gene ontology (GO) enrichment analysis for four main modules derived from Monocle 3. Figure S3. Identifying cell populations in the cerebellum with ST, related to Fig. 4. (a) Two cerebellar tissues of P7 WT mice were embedded in one slice for ST. (b) Mapping spots to their spatial positions shows that spots defined by marker genes are localized to the expected layers of cerebellum of sample II. Magnified images of the histological structures are shown in F5–F7. Scale bar: 25 μm. Figure S4. Identification and mapping of GN cell-type subpopulations across cerebellar regions, related to Fig. 5. (a) A more comprehensive classification of cell types in all samples. Cluster 3 is GNs I. (b) Feature plots of GNs I–specific genes. Figure S5. Developmental trajectories within GN lineage cells, related to Fig. 6. RNA velocities and the velocity-inferred root/end cells of Math1-GFP+ sorted samples at P [file 12915_2021_1071_MOESM1_ESM.zip › Fig S2 revised.tif]

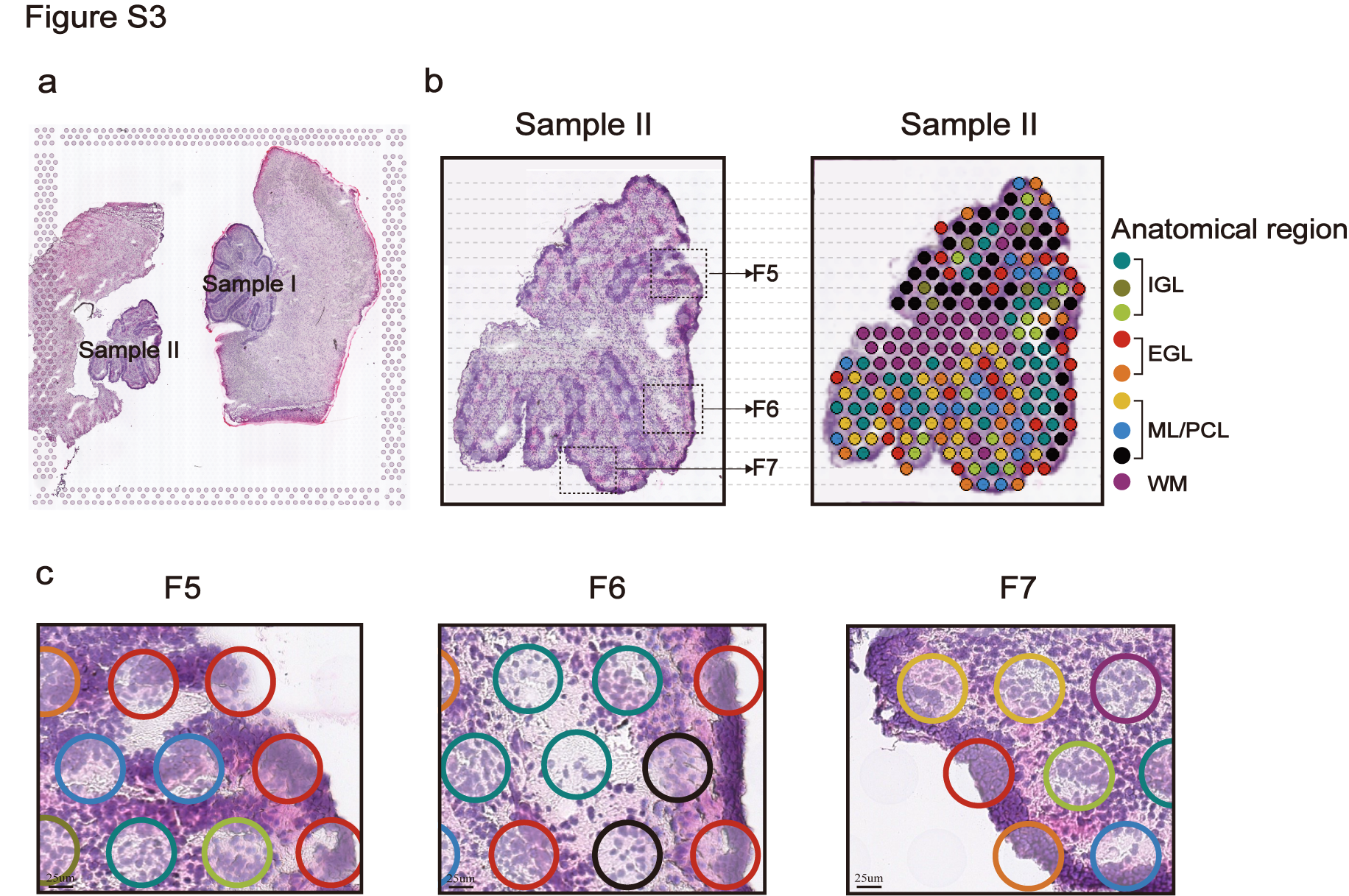

Supplement: Supplementary file 1 — Additional file 1: Figure S1. Single-cell transcriptome profiling of postnatal cerebellar cells, related to Fig. 1. (a) Workflow for cerebellum collection, single-cell sequencing and analysis of WT mice (one at P7 and the other at P11). (b) t-SNE visualization of 45,286 cells from four FACS-sorted samples integrated with two WT samples. Cells are colored according to clusters with annotation of cell types. (c) FACs data of Math1-GFP+, Dcx-DsRed+ strategies. (d) t-SNE visualization of 45,286 cells from FACS-sorted and WT samples (Math1-GFP+, Dcx-DsRed+ and WT cerebellum (CB)). (e) Left panel: Bar plots showing proportion of cell types in Math1-GFP+, Dcx-DsRed+ and WT samples. P < 0.0001. P values were determined using Pearson’s chi-square test. Right panel: Bar plot showing proportion of eight GNPs/GNs sub-clusters in Math1-GFP+, Dcx-DsRed+ and WT samples. (f) Signature genes scores of UBCs (Eomes) and PCs (Car8 and Calb1) in FACS-sorted samples. Figure S2. Identifying distinct states associated with postnatal GN development, related to Fig. 2. (a) Bar plots showing the results of gene ontology (GO) enrichment analysis for four main modules derived from Monocle 3. Figure S3. Identifying cell populations in the cerebellum with ST, related to Fig. 4. (a) Two cerebellar tissues of P7 WT mice were embedded in one slice for ST. (b) Mapping spots to their spatial positions shows that spots defined by marker genes are localized to the expected layers of cerebellum of sample II. Magnified images of the histological structures are shown in F5–F7. Scale bar: 25 μm. Figure S4. Identification and mapping of GN cell-type subpopulations across cerebellar regions, related to Fig. 5. (a) A more comprehensive classification of cell types in all samples. Cluster 3 is GNs I. (b) Feature plots of GNs I–specific genes. Figure S5. Developmental trajectories within GN lineage cells, related to Fig. 6. RNA velocities and the velocity-inferred root/end cells of Math1-GFP+ sorted samples at P [file 12915_2021_1071_MOESM1_ESM.zip › Fig S3 revised.tif]

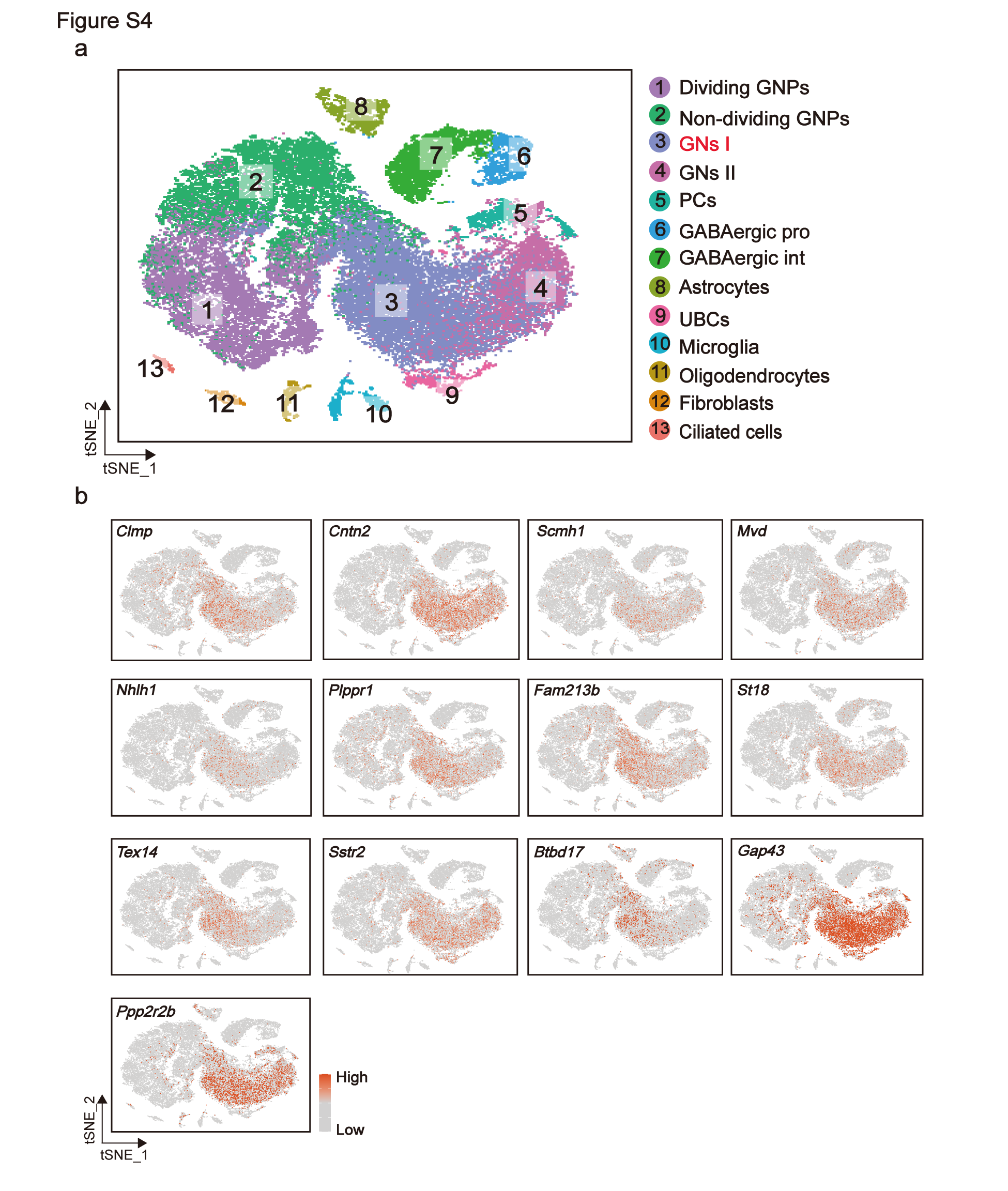

Supplement: Supplementary file 1 — Additional file 1: Figure S1. Single-cell transcriptome profiling of postnatal cerebellar cells, related to Fig. 1. (a) Workflow for cerebellum collection, single-cell sequencing and analysis of WT mice (one at P7 and the other at P11). (b) t-SNE visualization of 45,286 cells from four FACS-sorted samples integrated with two WT samples. Cells are colored according to clusters with annotation of cell types. (c) FACs data of Math1-GFP+, Dcx-DsRed+ strategies. (d) t-SNE visualization of 45,286 cells from FACS-sorted and WT samples (Math1-GFP+, Dcx-DsRed+ and WT cerebellum (CB)). (e) Left panel: Bar plots showing proportion of cell types in Math1-GFP+, Dcx-DsRed+ and WT samples. P < 0.0001. P values were determined using Pearson’s chi-square test. Right panel: Bar plot showing proportion of eight GNPs/GNs sub-clusters in Math1-GFP+, Dcx-DsRed+ and WT samples. (f) Signature genes scores of UBCs (Eomes) and PCs (Car8 and Calb1) in FACS-sorted samples. Figure S2. Identifying distinct states associated with postnatal GN development, related to Fig. 2. (a) Bar plots showing the results of gene ontology (GO) enrichment analysis for four main modules derived from Monocle 3. Figure S3. Identifying cell populations in the cerebellum with ST, related to Fig. 4. (a) Two cerebellar tissues of P7 WT mice were embedded in one slice for ST. (b) Mapping spots to their spatial positions shows that spots defined by marker genes are localized to the expected layers of cerebellum of sample II. Magnified images of the histological structures are shown in F5–F7. Scale bar: 25 μm. Figure S4. Identification and mapping of GN cell-type subpopulations across cerebellar regions, related to Fig. 5. (a) A more comprehensive classification of cell types in all samples. Cluster 3 is GNs I. (b) Feature plots of GNs I–specific genes. Figure S5. Developmental trajectories within GN lineage cells, related to Fig. 6. RNA velocities and the velocity-inferred root/end cells of Math1-GFP+ sorted samples at P [file 12915_2021_1071_MOESM1_ESM.zip › Fig S4 revised.tif]

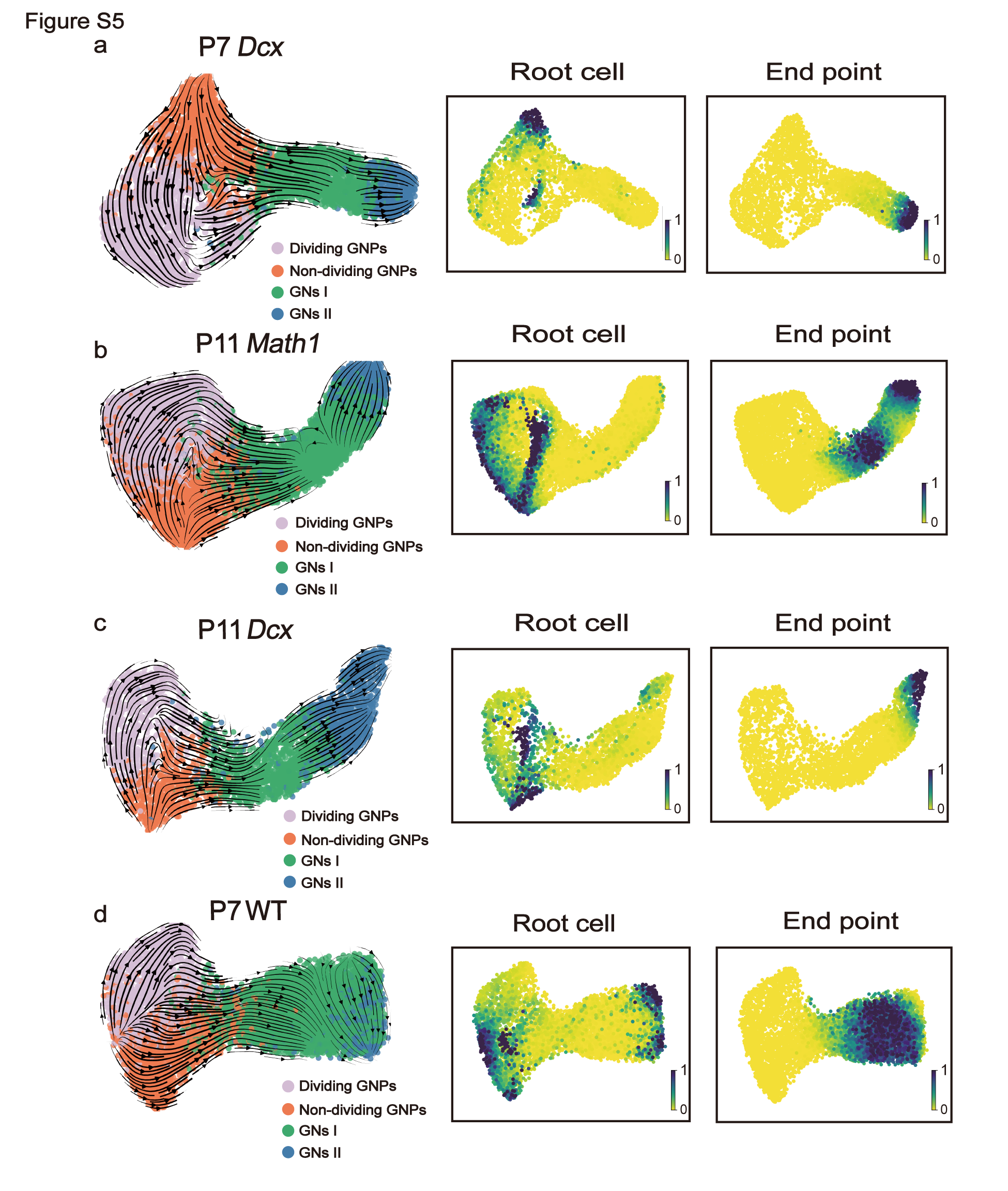

Supplement: Supplementary file 1 — Additional file 1: Figure S1. Single-cell transcriptome profiling of postnatal cerebellar cells, related to Fig. 1. (a) Workflow for cerebellum collection, single-cell sequencing and analysis of WT mice (one at P7 and the other at P11). (b) t-SNE visualization of 45,286 cells from four FACS-sorted samples integrated with two WT samples. Cells are colored according to clusters with annotation of cell types. (c) FACs data of Math1-GFP+, Dcx-DsRed+ strategies. (d) t-SNE visualization of 45,286 cells from FACS-sorted and WT samples (Math1-GFP+, Dcx-DsRed+ and WT cerebellum (CB)). (e) Left panel: Bar plots showing proportion of cell types in Math1-GFP+, Dcx-DsRed+ and WT samples. P < 0.0001. P values were determined using Pearson’s chi-square test. Right panel: Bar plot showing proportion of eight GNPs/GNs sub-clusters in Math1-GFP+, Dcx-DsRed+ and WT samples. (f) Signature genes scores of UBCs (Eomes) and PCs (Car8 and Calb1) in FACS-sorted samples. Figure S2. Identifying distinct states associated with postnatal GN development, related to Fig. 2. (a) Bar plots showing the results of gene ontology (GO) enrichment analysis for four main modules derived from Monocle 3. Figure S3. Identifying cell populations in the cerebellum with ST, related to Fig. 4. (a) Two cerebellar tissues of P7 WT mice were embedded in one slice for ST. (b) Mapping spots to their spatial positions shows that spots defined by marker genes are localized to the expected layers of cerebellum of sample II. Magnified images of the histological structures are shown in F5–F7. Scale bar: 25 μm. Figure S4. Identification and mapping of GN cell-type subpopulations across cerebellar regions, related to Fig. 5. (a) A more comprehensive classification of cell types in all samples. Cluster 3 is GNs I. (b) Feature plots of GNs I–specific genes. Figure S5. Developmental trajectories within GN lineage cells, related to Fig. 6. RNA velocities and the velocity-inferred root/end cells of Math1-GFP+ sorted samples at P [file 12915_2021_1071_MOESM1_ESM.zip › Fig S5 revised.tif]

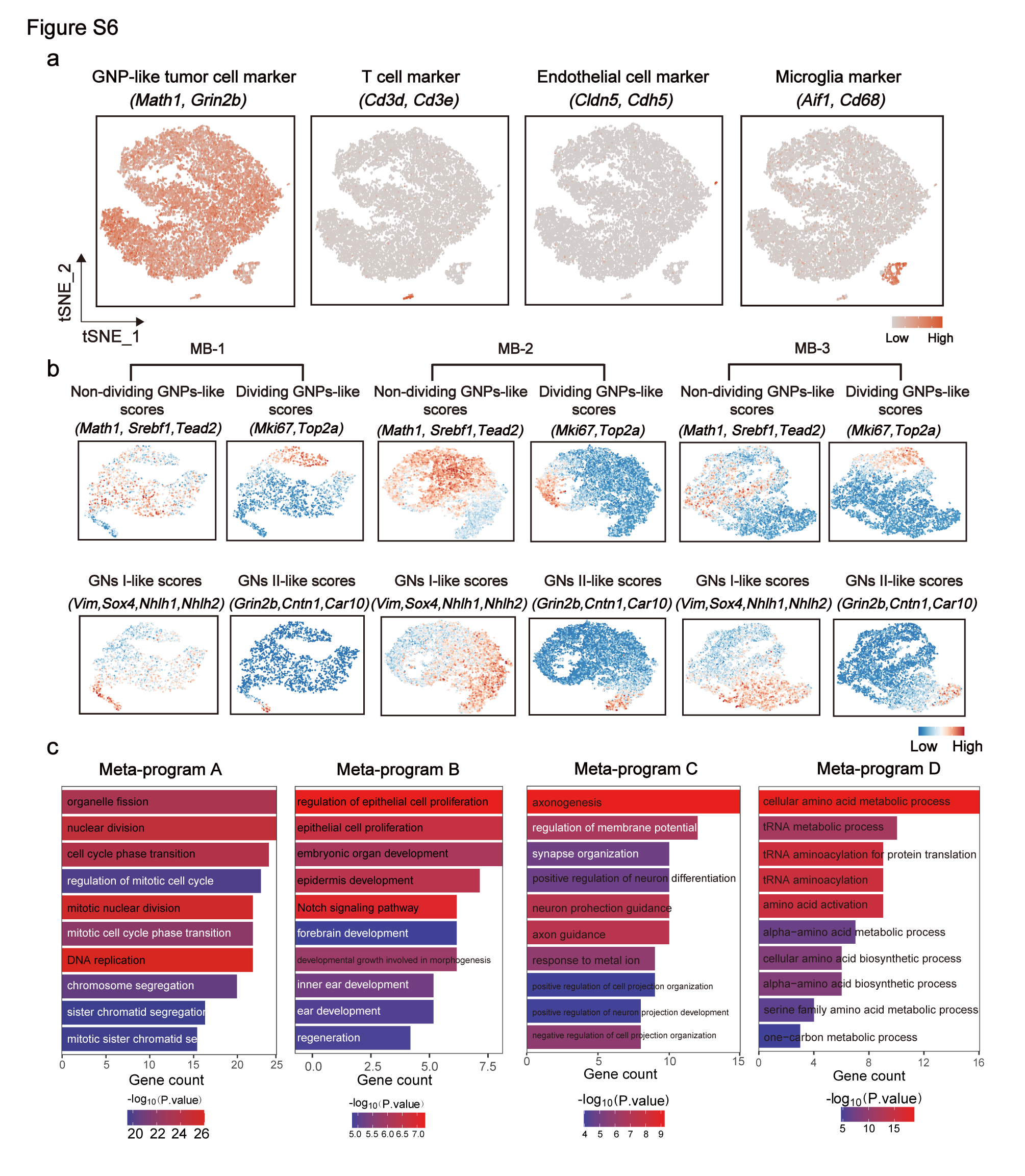

Supplement: Supplementary file 1 — Additional file 1: Figure S1. Single-cell transcriptome profiling of postnatal cerebellar cells, related to Fig. 1. (a) Workflow for cerebellum collection, single-cell sequencing and analysis of WT mice (one at P7 and the other at P11). (b) t-SNE visualization of 45,286 cells from four FACS-sorted samples integrated with two WT samples. Cells are colored according to clusters with annotation of cell types. (c) FACs data of Math1-GFP+, Dcx-DsRed+ strategies. (d) t-SNE visualization of 45,286 cells from FACS-sorted and WT samples (Math1-GFP+, Dcx-DsRed+ and WT cerebellum (CB)). (e) Left panel: Bar plots showing proportion of cell types in Math1-GFP+, Dcx-DsRed+ and WT samples. P < 0.0001. P values were determined using Pearson’s chi-square test. Right panel: Bar plot showing proportion of eight GNPs/GNs sub-clusters in Math1-GFP+, Dcx-DsRed+ and WT samples. (f) Signature genes scores of UBCs (Eomes) and PCs (Car8 and Calb1) in FACS-sorted samples. Figure S2. Identifying distinct states associated with postnatal GN development, related to Fig. 2. (a) Bar plots showing the results of gene ontology (GO) enrichment analysis for four main modules derived from Monocle 3. Figure S3. Identifying cell populations in the cerebellum with ST, related to Fig. 4. (a) Two cerebellar tissues of P7 WT mice were embedded in one slice for ST. (b) Mapping spots to their spatial positions shows that spots defined by marker genes are localized to the expected layers of cerebellum of sample II. Magnified images of the histological structures are shown in F5–F7. Scale bar: 25 μm. Figure S4. Identification and mapping of GN cell-type subpopulations across cerebellar regions, related to Fig. 5. (a) A more comprehensive classification of cell types in all samples. Cluster 3 is GNs I. (b) Feature plots of GNs I–specific genes. Figure S5. Developmental trajectories within GN lineage cells, related to Fig. 6. RNA velocities and the velocity-inferred root/end cells of Math1-GFP+ sorted samples at P [file 12915_2021_1071_MOESM1_ESM.zip › Fig S6 revised.tif]

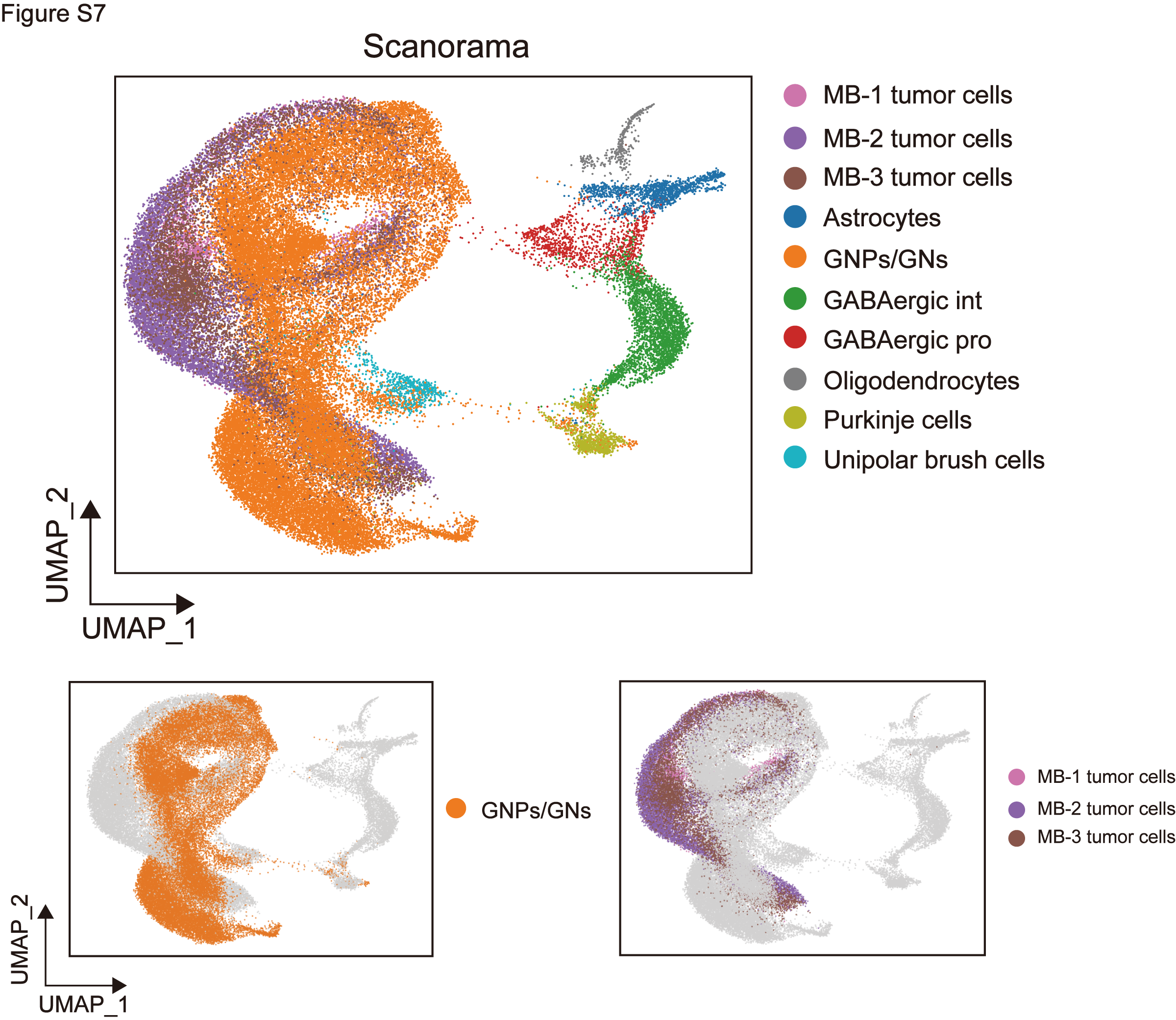

Supplement: Supplementary file 1 — Additional file 1: Figure S1. Single-cell transcriptome profiling of postnatal cerebellar cells, related to Fig. 1. (a) Workflow for cerebellum collection, single-cell sequencing and analysis of WT mice (one at P7 and the other at P11). (b) t-SNE visualization of 45,286 cells from four FACS-sorted samples integrated with two WT samples. Cells are colored according to clusters with annotation of cell types. (c) FACs data of Math1-GFP+, Dcx-DsRed+ strategies. (d) t-SNE visualization of 45,286 cells from FACS-sorted and WT samples (Math1-GFP+, Dcx-DsRed+ and WT cerebellum (CB)). (e) Left panel: Bar plots showing proportion of cell types in Math1-GFP+, Dcx-DsRed+ and WT samples. P < 0.0001. P values were determined using Pearson’s chi-square test. Right panel: Bar plot showing proportion of eight GNPs/GNs sub-clusters in Math1-GFP+, Dcx-DsRed+ and WT samples. (f) Signature genes scores of UBCs (Eomes) and PCs (Car8 and Calb1) in FACS-sorted samples. Figure S2. Identifying distinct states associated with postnatal GN development, related to Fig. 2. (a) Bar plots showing the results of gene ontology (GO) enrichment analysis for four main modules derived from Monocle 3. Figure S3. Identifying cell populations in the cerebellum with ST, related to Fig. 4. (a) Two cerebellar tissues of P7 WT mice were embedded in one slice for ST. (b) Mapping spots to their spatial positions shows that spots defined by marker genes are localized to the expected layers of cerebellum of sample II. Magnified images of the histological structures are shown in F5–F7. Scale bar: 25 μm. Figure S4. Identification and mapping of GN cell-type subpopulations across cerebellar regions, related to Fig. 5. (a) A more comprehensive classification of cell types in all samples. Cluster 3 is GNs I. (b) Feature plots of GNs I–specific genes. Figure S5. Developmental trajectories within GN lineage cells, related to Fig. 6. RNA velocities and the velocity-inferred root/end cells of Math1-GFP+ sorted samples at P [file 12915_2021_1071_MOESM1_ESM.zip › Fig S7 revised.tif]

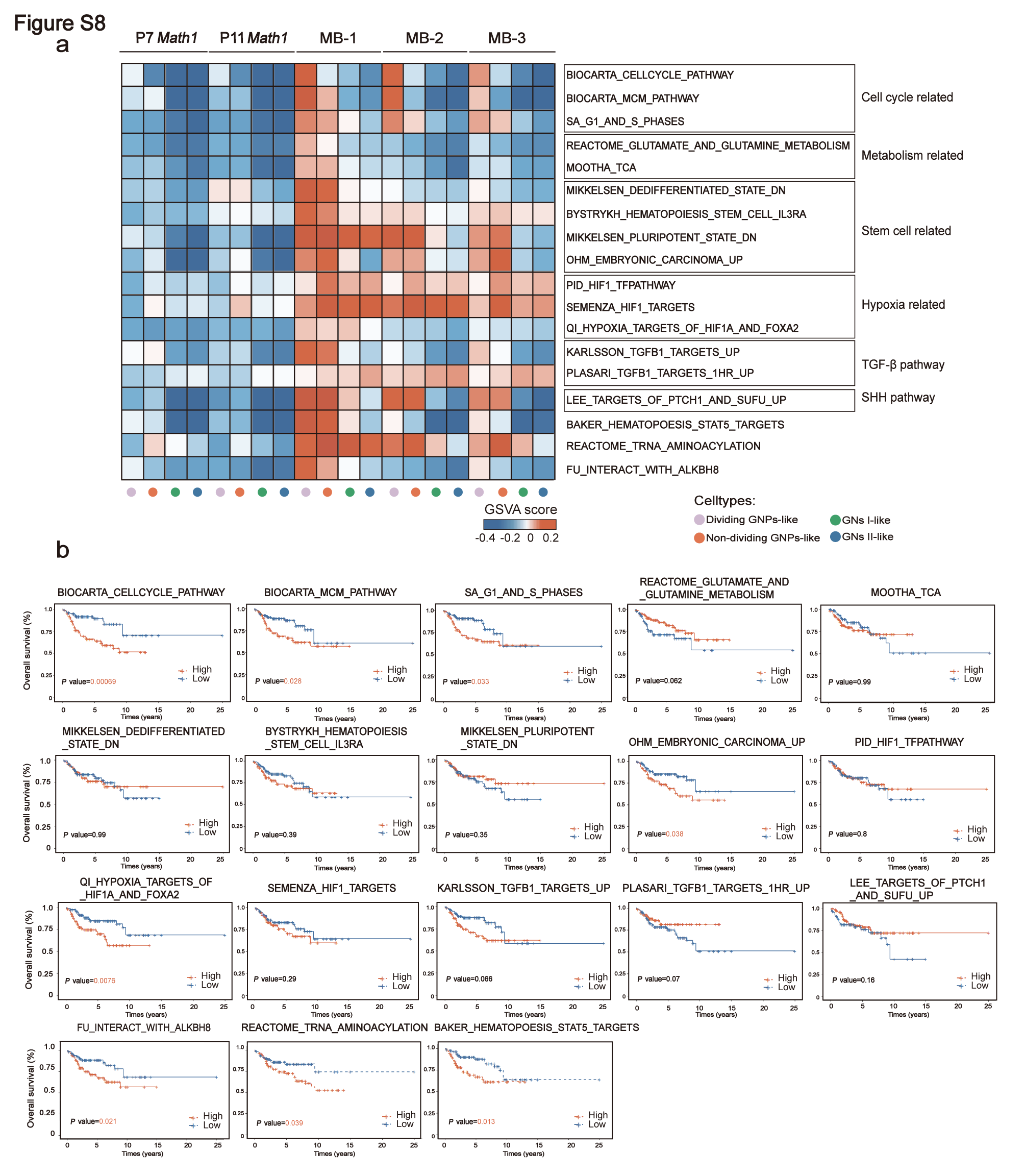

Supplement: Supplementary file 1 — Additional file 1: Figure S1. Single-cell transcriptome profiling of postnatal cerebellar cells, related to Fig. 1. (a) Workflow for cerebellum collection, single-cell sequencing and analysis of WT mice (one at P7 and the other at P11). (b) t-SNE visualization of 45,286 cells from four FACS-sorted samples integrated with two WT samples. Cells are colored according to clusters with annotation of cell types. (c) FACs data of Math1-GFP+, Dcx-DsRed+ strategies. (d) t-SNE visualization of 45,286 cells from FACS-sorted and WT samples (Math1-GFP+, Dcx-DsRed+ and WT cerebellum (CB)). (e) Left panel: Bar plots showing proportion of cell types in Math1-GFP+, Dcx-DsRed+ and WT samples. P < 0.0001. P values were determined using Pearson’s chi-square test. Right panel: Bar plot showing proportion of eight GNPs/GNs sub-clusters in Math1-GFP+, Dcx-DsRed+ and WT samples. (f) Signature genes scores of UBCs (Eomes) and PCs (Car8 and Calb1) in FACS-sorted samples. Figure S2. Identifying distinct states associated with postnatal GN development, related to Fig. 2. (a) Bar plots showing the results of gene ontology (GO) enrichment analysis for four main modules derived from Monocle 3. Figure S3. Identifying cell populations in the cerebellum with ST, related to Fig. 4. (a) Two cerebellar tissues of P7 WT mice were embedded in one slice for ST. (b) Mapping spots to their spatial positions shows that spots defined by marker genes are localized to the expected layers of cerebellum of sample II. Magnified images of the histological structures are shown in F5–F7. Scale bar: 25 μm. Figure S4. Identification and mapping of GN cell-type subpopulations across cerebellar regions, related to Fig. 5. (a) A more comprehensive classification of cell types in all samples. Cluster 3 is GNs I. (b) Feature plots of GNs I–specific genes. Figure S5. Developmental trajectories within GN lineage cells, related to Fig. 6. RNA velocities and the velocity-inferred root/end cells of Math1-GFP+ sorted samples at P [file 12915_2021_1071_MOESM1_ESM.zip › Fig S8 revised.tif]
